# Recent advances in synthesis and medicinal chemistry of pyrrolodiazepines

**DOI:** 10.3389/fchem.2026.1893737

**Published:** 2026-08-12

**Authors:** Mengyang Zhou, Shutao Wang

**Affiliations:** 1 Department of Pharmacy, China-Japan Union Hospital of Jilin University, Changchun, China; 2 Department of Pharmacy, The Second Hospital of Jilin University, Changchun, China

**Keywords:** biological activity, drug discovery, heterocyclic chemistry, pyrrolodiazepines, synthesis

## Abstract

Nitrogen-containing heterocyclic compounds represent fundamental structural elements in contemporary medicinal chemistry. Pyrrolodiazepines, which result from the fusion of pyrrole and diazepine rings, integrate the beneficial properties of both parent structures and have emerged as a significant area of research in synthetic chemistry and pharmaceutical development in recent years. This review provides a systematic overview of the synthetic methodologies for pyrrolodiazepine derivatives that have been developed recently, encompassing transition-metal catalysis, cascade cyclization, multicomponent reactions, photoredox catalysis, base-mediated cyclization, and environmentally friendly synthesis approaches. The advantages and limitations of each method are critically evaluated. Furthermore, the biological activities of pyrrolodiazepines are extensively reviewed, addressing their antitumor, antibacterial, antiviral, central nervous system regulatory, metabolic, and cardiovascular effects, along with corresponding structure–activity relationship analyses. Finally, the review identifies the current challenges in the synthesis and application of pyrrolodiazepines and explores potential future development directions.

## 1 Introduction

Nitrogen-containing heterocyclic frameworks are fundamental to contemporary medicinal chemistry, forming the core structural component of over 80% of clinically approved small-molecule drugs (Ebenezer et al., 2022; Kumar et al., 2023; Marshall et al., 2024). Within this category, diazepines have gained prominence as one of the most pharmacologically versatile chemotypes. Three major diazepine subtypes, including 1,4-diazepines, 1,3-diazepines and 1,2-diazepines, act as key scaffolds for therapeutic agents targeting multiple diseases. These scaffolds are widely applied to treat central nervous system (CNS) disorders, tumors, infectious diseases and inflammatory conditions. (Beke et al., 2026; Ren et al., 2025; You et al., 2026; Zhou et al., 2026). The exceptional biological efficacy of diazepine derivatives can be attributed to their distinctive conformational flexibility, hydrogen-bonding capacity, and their ability to interact with a diverse array of biological targets, including G protein-coupled receptors (GPCRs), enzymes, ion channels, and DNA.

**FIGURE 1:**
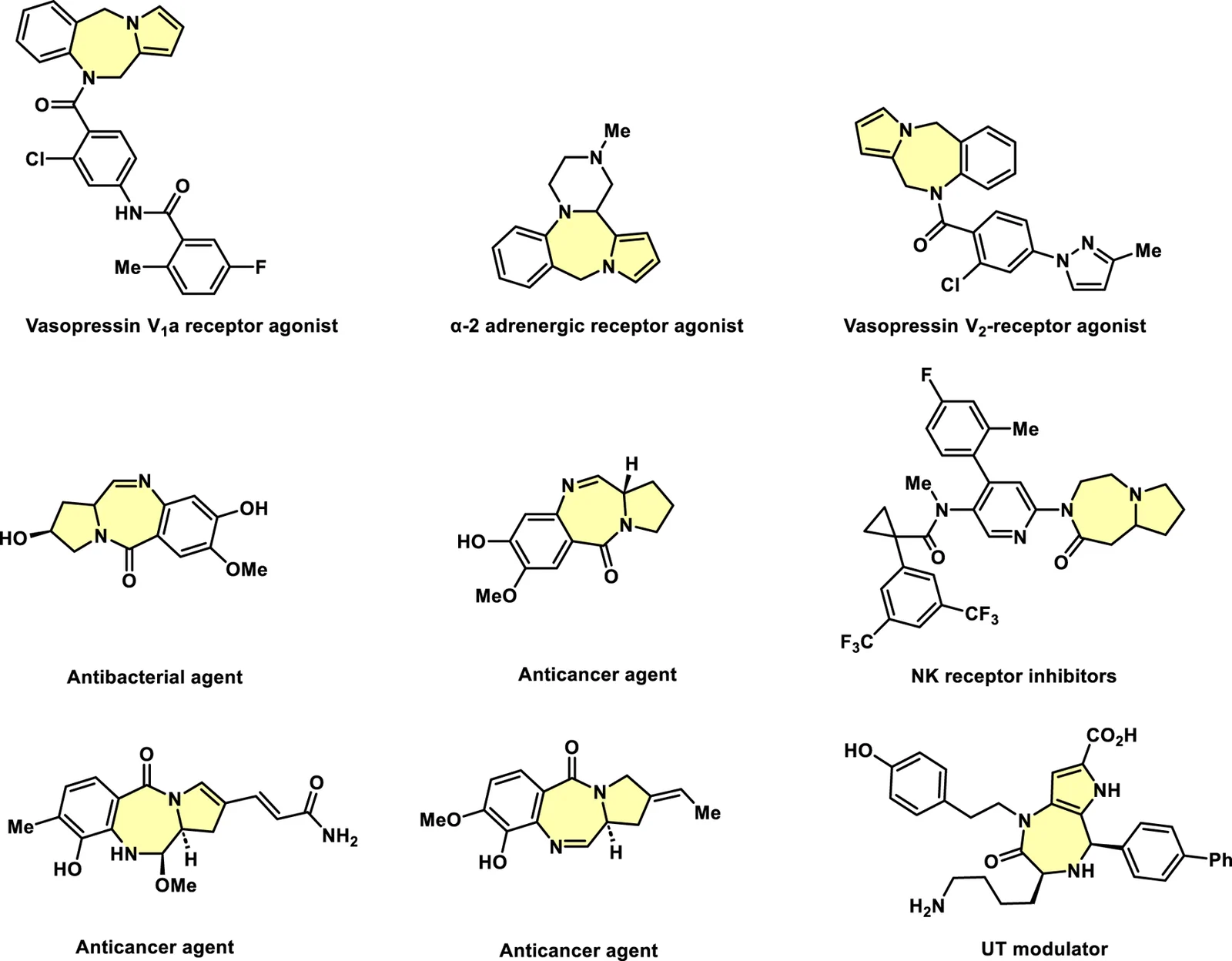
Bioactive molecules containing pyrrolodiazepine frameworks.

Pyrrole is a five-membered aromatic heterocycle widely found in natural products, bioactive alkaloids and drug molecules (Hunjan et al., 2021; Mateev et al., 2022; Pramanik et al., 2023). It endows molecules with planar aromatic structures and electron-rich π-conjugation, alongside favorable physicochemical properties, thereby enriching compound pharmacological performance. Diazepine is a seven-membered nitrogen heterocycle and a well-recognized privileged medicinal scaffold (Arora et al., 2020; Leśniewska and Przybylski, 2024; Malki et al., 2021; Muhammad et al., 2019). It balances moderate rigidity and flexible conformations, and displays potent bioactivities, especially CNS regulatory and anti-tumor effects. Chemists can fuse pyrrole and diazepine rings to construct a unique family of tricyclic heterocycles named pyrrolodiazepines (Figure 1). This fused scaffold integrates the structural and pharmacological merits of both pyrrole and diazepine (Dufour-Gallant et al., 2015; Lee et al., 2025; Saquib et al., 2023; Shi et al., 2021). The fused ring system increases molecular rigidity, which improves target binding affinity and selectivity. It also expands the accessible chemical space for drug screening, enabling the creation of new scaffolds with novel action mechanisms and better druggability.

**FIGURE 2:**
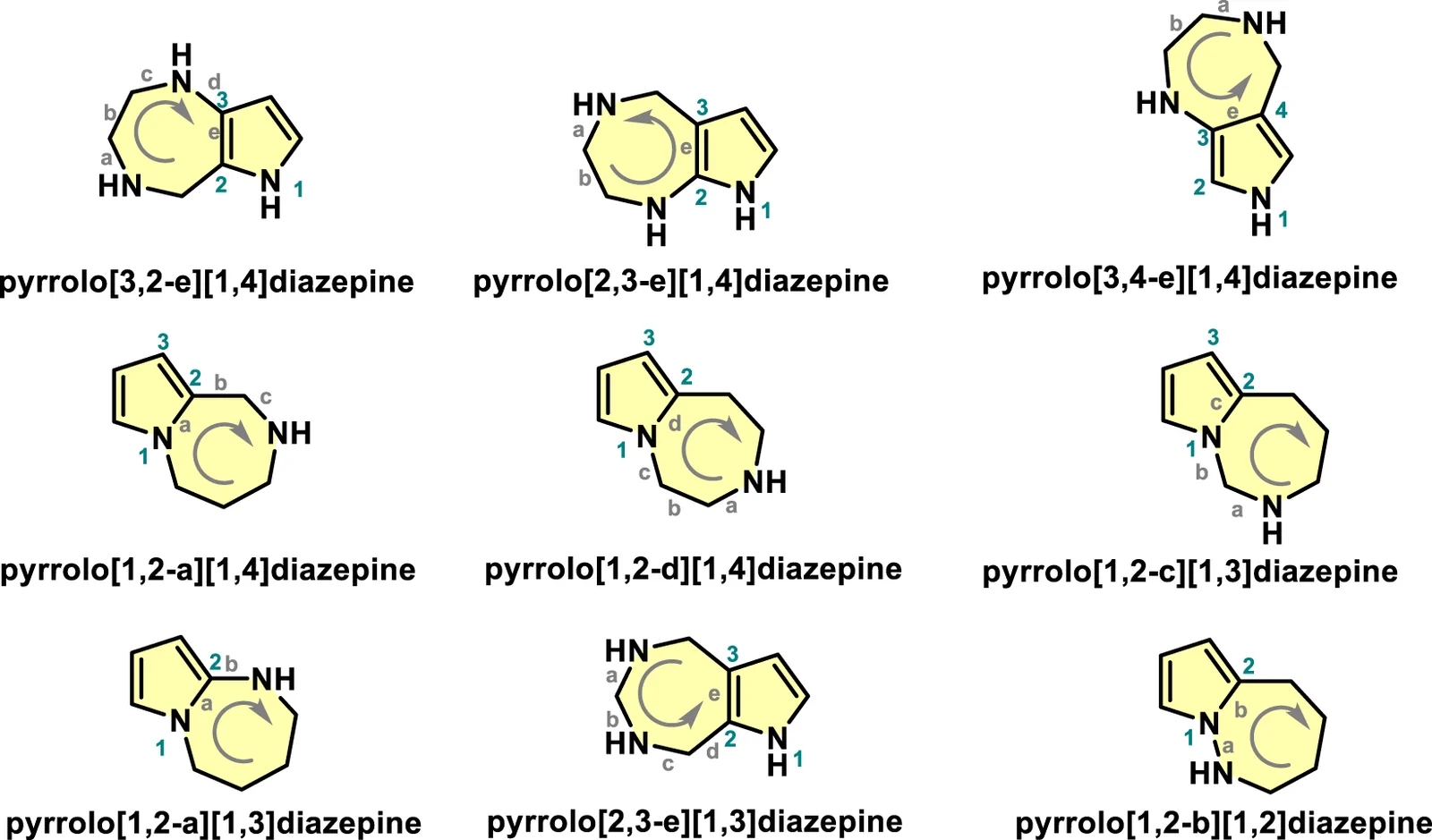
Diverse regioisomeric frameworks of pyrrole-fused diazepines.

Over the past 5 years, pyrrolodiazepines have garnered increasing interest from synthetic and medicinal chemists globally (Chen et al., 2023; Gu et al., 2023; Shawky et al., 2021). Various regioisomeric frameworks have been synthesized, including C–C fused pyrrolo[3,2-e][1,4]diazepines, pyrrolo[2,3-e][1,4]diazepines, pyrrolo[3,4-e][1,4]diazepines, and N–C fused pyrrolo[1,2-d][1,4]diazepines, pyrrolo[1,2-a][1,4]diazepines, among others. Nevertheless, not all theoretically possible pyrrole-fused diazepine regioisomers have been synthesized. Consequently, this review focuses solely on those variants that have been experimentally validated (Figure 2). Different regioisomers differ drastically in spatial geometry and electronic distribution. Such structural differences lead to diverse biological functions, covering anti-tumor, antibacterial, antiviral and anti-parasitic activity. They also act as CNS modulators, enzyme inhibitors, or receptor agonists/antagonists.

Despite considerable progress, the development of pyrrolodiazepine-based therapeutics continues to encounter significant challenges. These include limited synthetic methodologies for regioselective ring construction, a poor understanding of structure–activity relationships (SAR), and insufficient exploration of less-studied regioisomers. Traditional synthetic approaches often involve harsh reaction conditions, multistep procedures, low atom economy, and limited substrate compatibility, which hinder the rapid construction of compound libraries necessary for high-throughput screening. The SAR of pyrrolodiazepines remains inadequately characterized, with limited studies systematically addressing the influence of fusion patterns, substituent types, and stereochemistry on target interaction, potency, and selectivity. Our research team has long focused on designing and synthesizing fused diazepine heterocycles. We have constructed a series of indole- and pyrrole-diazepine hybrid scaffolds containing bridgehead nitrogen atoms. These studies offer practical references for the synthetic and medicinal chemistry of this privileged scaffold class (Wang et al., 2021; Wang et al., 2025; Zhou et al., 2026).

To better highlight the novelty and incremental value of the present work, we systematically summarize the coverage and limitations of previously published review articles related to pyrrolodiazepines and fused diazepine heterocycles. Early reviews on diazepine scaffolds mainly focused on benzodiazepines and simple 1,4-or 1,3 diazepines, with limited discussion of pyrrole-fused tricyclic derivatives (Laura et al., 2009; Muhammad et al., 2019; Smith et al., 2014; Tamas et al., 2018). Several recent heterocyclic chemistry reviews briefly touched on partial pyrrolodiazepine synthetic routes, yet they only covered single reaction types (e.g., metal-free cyclization or Pd-catalyzed C–H activation) without systematically categorizing all mainstream cyclization strategies, including photoredox catalysis, organocatalytic [4+3] annulation, biomass-mediated green synthesis, and enantioselective asymmetric cyclization (Jaiswal et al., 2025; Malki et al., 2022; Pranshu and Navjeet, 2023; Saranya et al., 2021; Sonali et al., 2023).

From a pharmacological perspective, existing summaries only scatteredly reported the anticancer or antibacterial activities of individual pyrrolodiazepine analogs, lacking a comprehensive cross-domain collation of central nervous system regulatory, antiviral, cardiovascular and metabolic regulatory effects. More importantly, no prior review has systematically correlated ring fusion topology (C–C fused vs. N–C fused), conformational dynamics, substituent steric/electronic effects and stereochemistry to construct an integrated SAR framework for all pyrrolodiazepine regioisomers. Additionally, underexplored subtypes including 1,3- and 1,2-diazepine-fused pyrroles have long been ignored in past reviews, and few literature discussed photoswitchable pyrrolodiazepine photopharmacological agents and chiral pyrrolodiazepine synthetic advances emerging in the past 5 years.

This review aims to provide a comprehensive overview of recent progress in pyrrolodiazepine chemistry over the past 5 years. The discussion focuses on two key research directions. On one hand, it highlights the development of versatile synthetic strategies for constructing diverse pyrrolodiazepine scaffolds, including metal-catalyzed annulation, cascade/domino cyclization, multicomponent reactions, photoredox catalysis, and base-mediated cyclization. On the other hand, it explores the therapeutic potential and biological activities of pyrrolodiazepine derivatives, covering anticancer, antimicrobial, antiviral, central nervous system regulatory, and enzyme inhibitory effects, accompanied by in-depth analyses of SAR. By sorting representative synthetic protocols, summarizing bioactive lead compounds, and analyzing existing limitations and future research trends, this work provides a complete reference for organic and medicinal chemists. It supports rational molecular design, efficient scaffold synthesis and translational research of pyrrolodiazepine candidate drugs.

## 2 Synthetic strategies

Pyrrolodiazepines, as a class of privileged nitrogen-containing heterocyclic scaffolds, have demonstrated significant potential in the treatment of central nervous system disorders, malignant tumors, and drug-resistant microbial infections. Given the rising prevalence of multidrug resistance and the unmet clinical need for target-selective agents, the development of efficient and modular synthetic routes for structurally diverse pyrrolodiazepines has become a critical focus in contemporary organic and medicinal chemistry. Consequently, substantial efforts have been directed toward establishing versatile synthetic methodologies that allow precise control over regioselectivity, stereochemistry, and functional group compatibility. The following sections systematically discuss advanced synthetic strategies for constructing pyrrole-fused diazepine frameworks. Particular emphasis is placed on 1,4-diazepine-fused pyrrole derivatives, as they represent the most extensively studied and biologically promising subclass.

### 2.1 Methods for the synthesis of 1,4-diazepines fused pyrrole

The strategic construction of pyrrolodiazepine frameworks necessitates precise control over ring fusion topology, regioselectivity, and functional group compatibility, which has spurred the development of versatile and modular synthetic methodologies over the past decade. Pyrrolodiazepines, as a class of structurally unique tricyclic heterocycles resulting from the fusion of pyrrole and diazepine units, exhibit multiple regioisomeric connection modes, including C–C fused and N–C fused subtypes, each necessitating distinct synthetic strategies for their efficient and selective construction. To date, a variety of synthetic methodologies have been developed to access these privileged scaffolds. These methodologies encompass transition-metal-catalyzed annulations, cascade/domino cyclizations, base-mediated transformations, photoredox catalysis, and multicomponent reactions. These approaches overcome the intrinsic challenges of seven-membered diazepine ring formation, including entropic penalties and transannular interactions. Moreover, they enable facile diversification of pyrrolodiazepine cores with various substituents, thereby providing a robust platform for structure–activity relationship studies and therapeutic exploration.

In this section, we systematically classify and summarize the advanced synthetic strategies for pyrrolodiazepines, organized by the isomerism of the diazepine ring and fusion patterns (C–C versus N–C). For each subtype, we present representative protocols, detail the reaction mechanisms, and discuss substrate scope and selectivity control. The advantages and limitations of each approach are also evaluated to provide a practical guide for synthesizing structurally diverse pyrrolodiazepine derivatives.

#### 2.1.1 C–C fused

C–C fused pyrrolodiazepines constitute a significant class of regioisomerically defined heterocyclic frameworks, characterized by the linkage of pyrrole and diazepine rings through direct carbon–carbon bonds. This fusion imparts the molecules with a more planar conformation and enhanced π conjugation, rendering them particularly adept at interacting with planar biological targets such as kinases, nucleic acids, and aromatic binding pockets. Among the diverse C–C fused isomers, pyrrolo[3,2-e][1,4]diazepines are the most extensively investigated and structurally well-characterized subclass, exhibiting notable anticancer activities through the inhibition of key oncogenic kinases. The following provides a detailed introduction to the synthetic strategies for this crucial scaffold.

##### 2.1.1.1 Pyrrolo[3,2-e][1,4]diazepine

**SCHEME 1:**
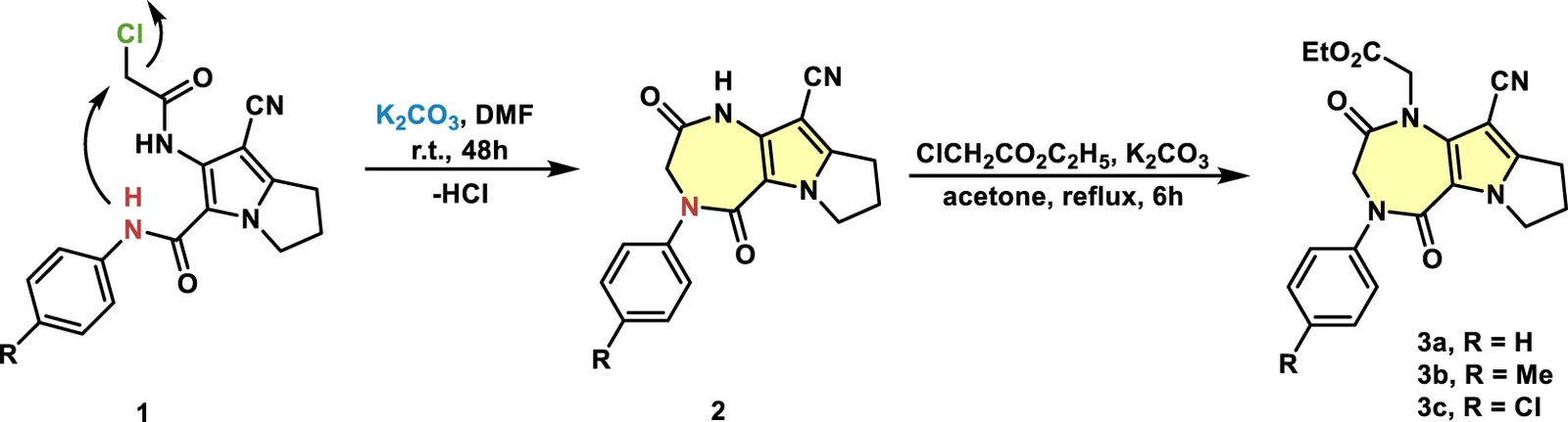
Synthesis of EGFR/CDK2 inhibitors.

Pyrrolo[3,2-e][1,4]diazepines constitute a significant class of regioselectively fused heterocyclic frameworks with notable inhibitory activity against EGFR and CDK2, rendering them promising candidates for antitumor drug development. Within this category, the ethyl ester analogues **3a–c** have been synthesized through a streamlined two-step synthetic approach, beginning with the cyano-substituted precursors **1** (Scheme 1) (Belal et al., 2022). Initially, the pivotal diazepino [5,6-b]pyrrolizine-10-carbonitrile intermediates **2** were generated via intramolecular cyclization of compounds **1**. Compounds **1** bear intramolecular nucleophilic amide nitrogen and a chloromethyl leaving group. Upon deprotonation mediated by weak base potassium carbonate, intramolecular S_N_2 cyclization proceeds with the elimination of chloride ions, affording the pharmaceutically privileged fused seven-membered 1,4-diazepine heterocycle. This cyclization was effectively facilitated by potassium carbonate in DMF under mild conditions. Following the successful formation of the cyclic core structures, compounds **3a–c** were synthesized through *N*-alkylation of intermediates **2a–c** using ethyl chloroacetate. The alkylation process was conducted in acetone with anhydrous potassium carbonate as the base, and the desired ethyl ester derivatives were isolated after refluxing for 6 h.

##### 2.1.1.2 Pyrrolo[2,3-e][1,4]diazepine

**SCHEME 2:**
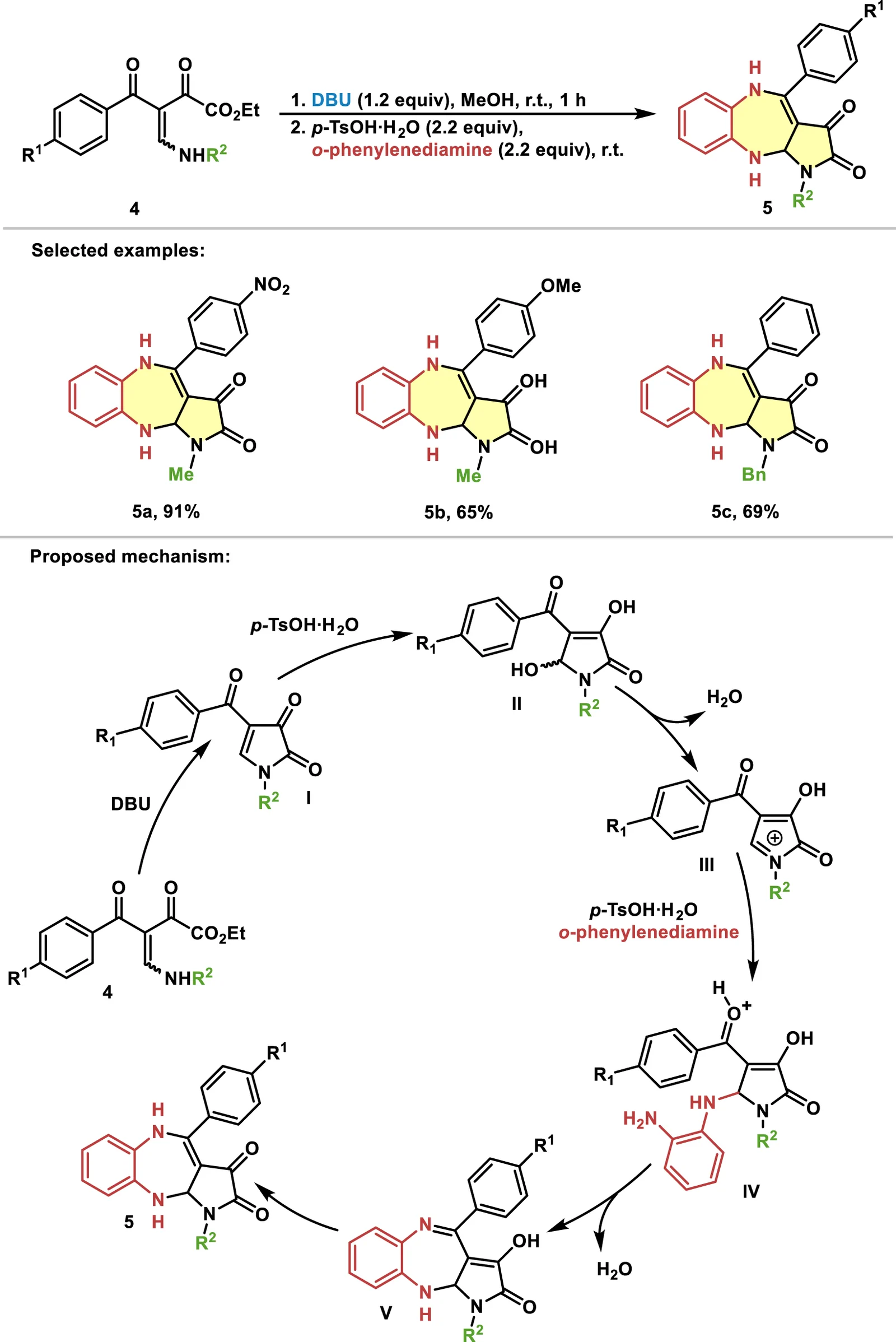
Synthesis and proposed mechanism of pyrrole-fused 1,5 benzodiazepines 5 via metal-free cascade cyclization/annulation of β-enamino diketones with *o*-phenylenediamine.

Among the regioisomeric pyrrolo[2,3-e][1,4]diazepine frameworks, the efficient and selective construction of the core skeleton remains a primary objective for medicinal chemists. To meet this demand, a mild and scalable synthetic methodology has been developed for the synthesis of structurally related pyrrole-fused 1,5-benzodiazepine analogs. Poletto et al. introduced a cascade cyclization/annulation strategy that facilitates the rapid synthesis of pyrrole-fused 1,5-benzodiazepines **5** via a one-pot, metal-free process (Scheme 2) (Poletto et al., 2024). This approach utilizes β-enamino diketones (BEDs) as versatile C–C–C synthons and o-phenylenediamine as a bifunctional nucleophile, achieving excellent chemo- and regioselectivity under mild reaction conditions. Initially, the β-enamino diketone precursor four is dissolved in methanol and treated with DBU, which promotes rapid intramolecular cyclization to form the 4-acylpyrrole-2,3-dione intermediate within 1 h. Subsequently, *p*-TsOH·H_2_O and *o*-phenylenediamine (OPD) are directly added to the same reaction vessel. This acidic environment facilitates the formation of the key *N*-acyliminium ion, which undergoes immediate annulation with *o*-phenylenediamine. The reaction is allowed to proceed for 2 h for *N*-methyl substrates, or 12 h for more sterically hindered *N*-benzyl or *N*-aryl derivatives. Upon completion of the reaction, the target pyrrolo[5,4-b][1,5]benzodiazepine products five precipitate from the reaction mixture and are subsequently isolated through straightforward filtration, followed by washing with cold methanol and vacuum drying.

The divergent reactivity between primary and secondary BEDs with OPD constitutes the cornerstone of this cascade strategy. Primary BEDs (*R* ^2^ = H) undergo direct cyclocondensation with OPD at the α-oxo ester moiety, furnishing six-membered quinoxalinones as the by-products. In stark contrast, secondary BEDs (**4**, *R* ^2^ = Me, Bn, or Ar) are sterically and electronically precluded from this pathway. The *N*-substituent on the enamine nitrogen effectively blocks the 1,2-dielectrophilic center, thereby redirecting the reaction course toward an intramolecular cyclization manifold. This process generates a 4-acyl-1*H*-pyrrole-2,3-dione intermediate **I**, which, upon acid activation, forms an *N*-acyliminium species **III** that is subsequently captured by OPD. The resulting intermediate undergoes a kinetically favorable seven-*exo*-trig cyclization, ultimately delivering the pyrrolo[5,4-*b*][1,5]benzodiazepine scaffold **5** with high chemo- and regioselectivity. This substituent-controlled switch between [4+2] and cascade [3+2+2] annulation modes enables programmable access to either quinoxalinone or fused benzodiazepine architectures from a common BED precursor.

Electron-withdrawing aryl groups and small *N*-alkyl substituents (Me, Bn) afforded high yields up to 91%, showing good functional group compatibility for simple scaffolds. Nevertheless, yield analysis exposes clear drawbacks: *N*-aryl substrates capped at 80% (mostly 69%–78%) due to steric inhibition of the seven-exo-trig cyclization, hampering access to pharmaceutically relevant *N*-aromatic analogues. Electron-rich 4-methoxyphenyl substituents consistently lowered yields by 10%–25%; *N*-methyl substrates completed reactions within 2 h, whereas *N-*benzyl and *N*-aryl substrates required stirring for up to 12 h, lowering overall synthetic consistency for scale-up. High yields (>90%) were exclusive to electron-poor aroyl/*N*-alkyl combinations, while bis-electron-rich aromatic frameworks gave yields below 70%.

##### 2.1.1.3 Pyrrolo[3,4-e][1,4]diazepine

**SCHEME 3:**
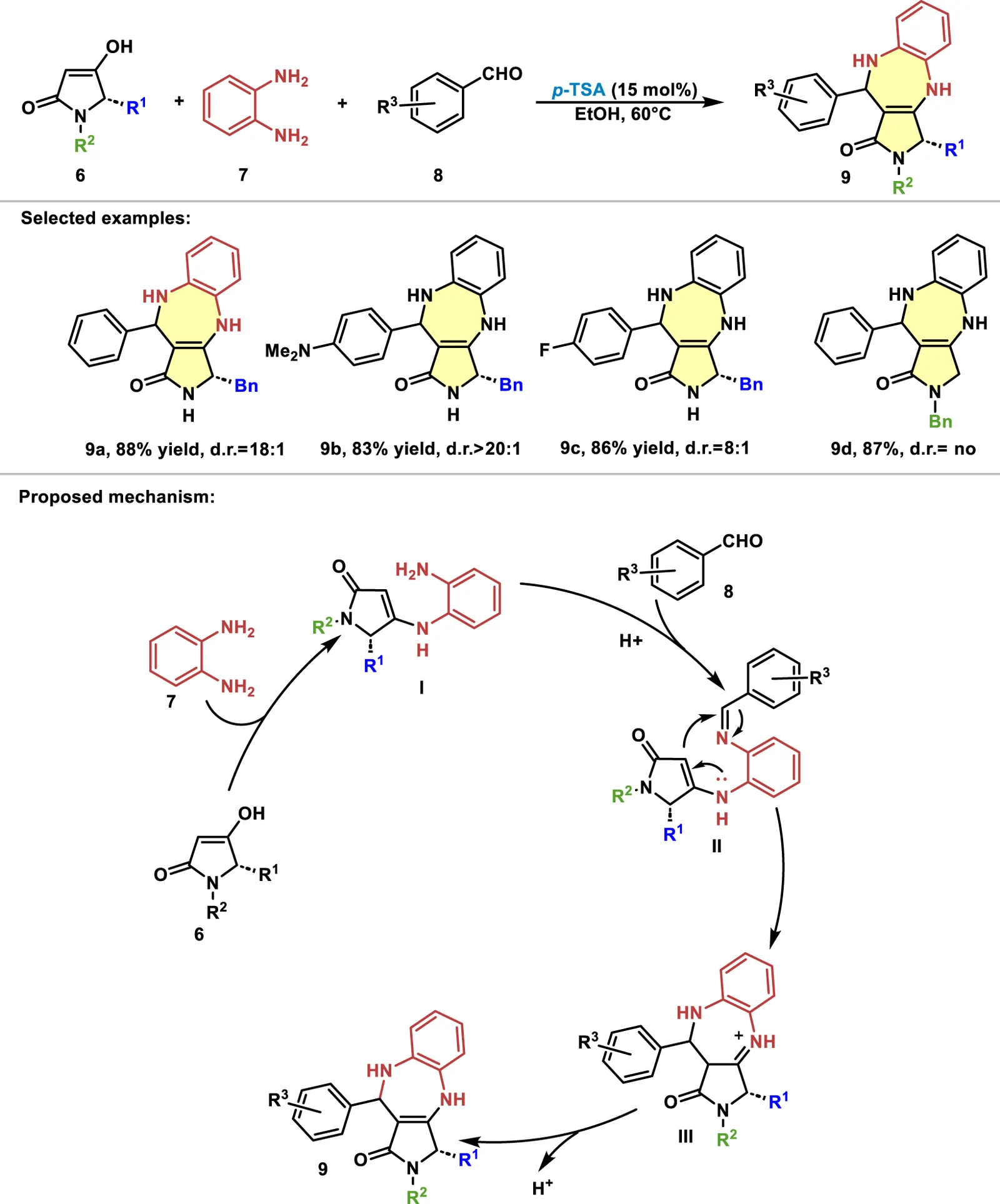
Synthesis and proposed mechanism of 1,5-benzodiazepines with pyrrolidinone motifs via *p*-TSA-catalyzed one-pot three-component reaction.

In addition to these regioisomers, the pyrrolo[3,4-e][1,4]diazepine framework constitutes a valuable fused scaffold with promising biological profiles. A series of novel 1,5-benzodiazepine derivatives incorporating pyrrolidinone motifs nine were synthesized via an efficient, environmentally benign one-pot three-component reaction (Scheme 3). This reaction utilized tetramic acids **6**, 1,2-phenylenediamine **7**, and aromatic aldehydes **8** as starting materials (Han et al., 2021). Under optimized conditions, the reaction proceeded efficiently with *p*-toluenesulfonic acid (*p*-TSA, 15 mol%) serving as the catalyst in ethanol at 60 °C for 3 h, yielding the desired compounds in high isolated yields (80%–88%) with excellent diastereoselectivities (up to >20:1 d.r.). This multicomponent strategy offers significant advantages, including operational simplicity, mild reaction conditions, and facile purification, as the products precipitate directly from the reaction mixture upon cooling, thereby reducing waste generation.

The reaction progresses through a one-pot, three-component cascade mechanism. Initially, 1,2-phenylenediamine seven undergoes condensation with tetramic acid **6**, resulting in the formation of an enaminone intermediate **I**. This intermediate subsequently engages in a reaction with an aromatic aldehyde eight under acid catalysis, leading to the generation of an iminium intermediate **II**. This pivotal intermediate undergoes an intramolecular Michael addition, followed by proton transfer and isomerization, ultimately yielding the fused 1,5-benzodiazepine framework nine incorporating a pyrrolidinone ring.

The three-component reaction exhibits excellent diastereoselectivity, affording benzodiazepine derivatives with diastereomeric ratios ranging from 8:1 to >20:1. The stereochemical outcome is governed by substrate-controlled asymmetric induction originating from the *(S)*-configured tetramic acid. The benzyl group at C3 of the pyrrolidinone ring creates a sterically biased environment that directs the intramolecular Michael addition to occur preferentially from the Si-face of the imine intermediate, leading to the formation of the (3S,10S)-configured product as the major diastereomer.

The electronic nature of the aromatic aldehyde substituent (R^3^) significantly influences the diastereoselectivity. Electron-donating groups (e.g., OMe, NMe_2_) at the para or meta positions furnish superior selectivities (d.r. > 20:1), presumably due to attenuated iminium electrophilicity that increases reaction reversibility and favors thermodynamic control. Conversely, electron-withdrawing groups (e.g., Br, F) diminish the selectivity to 8:1–9:1, suggesting a shift toward kinetic control. This one-pot multicomponent reaction achieves excellent control over diastereoselectivity, chemoselectivity and regioselectivity through a combination of substrate-controlled asymmetric induction, strategic stepwise addition, and optimized reaction conditions, providing a practical and atom-economical approach to enantiomerically enriched 1,5-benzodiazepine derivatives containing a pyrrolidinone motif.

#### 2.1.2 N–C fused

In contrast to C–C fused analogues, N–C fused pyrrolodiazepines feature a ring-fusion mode that connects the pyrrole and diazepine cores through a shared nitrogen atom, endowing the scaffold with unique three-dimensional folded conformations and distinct target-binding preferences. This structural characteristic makes N–C fused derivatives particularly promising for modulating GPCRs, enzymes, and other therapeutic targets associated with central nervous system disorders and antimicrobial activities. Among all N–C fused regioisomers, pyrrolo[1,2-a][1,4]diazepine is the most well-developed and versatile subclass, with a wealth of efficient synthetic strategies established for its construction.

##### 2.1.2.1 Pyrrolo[1,2-a][1,4]diazepine

**SCHEME 4:**
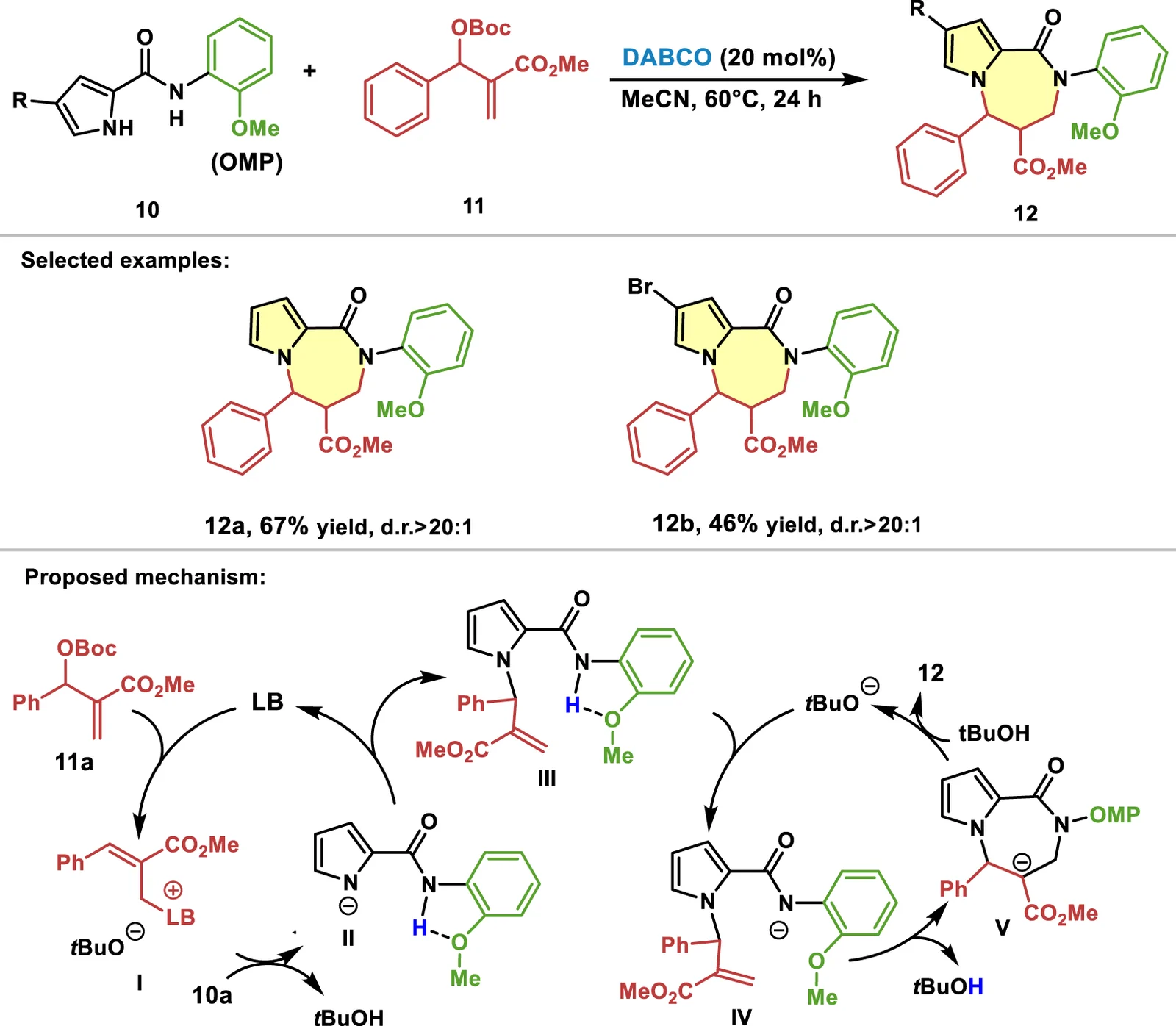
Synthesis and proposed mechanism of pyrrolo[1,2-a][1,4]diazepine derivatives 12 via OMP-directed [4 + 3] Annulation.

In addition to the C–C fused pyrrolodiazepine frameworks, the N–C fused analogues constitute another significant subclass, characterized by distinct structural features and promising biological activities. Notably, pyrrolo[1,2-a][1,4]diazepine has garnered considerable attention due to its unique ring-fusion mode and versatile pharmacological properties. The design and synthesis of compounds **12a** and **12b** were achieved via a Lewis base-catalyzed [4 + 3] annulation reaction between pyrrole-2-carboxamides, which possess the OMP directing group, and MBH carbonates, following optimized conditions with 20 mol% DABCO as the catalyst in MeCN (Scheme 4) (Wang et al., 2025). Owing to the comparatively lower reactivity of pyrrole substrates relative to indoles, these reactions necessitated elevated temperatures (60 °C) and prolonged reaction times (24 h), resulting in yields of 67% for compound **12a** and 46% for **12b**.

The OMP directing group is instrumental in the annulation process by facilitating precise control over regioselectivity. This is achieved through the formation of an intramolecular hydrogen bond between its ortho-methoxy group and the amide NH group of the pyrrole-2-carboxamide, which stabilizes the amide conformation and diminishes the nucleophilicity of the amide NH. This interaction preferentially promotes the initial nucleophilic attack by the more reactive pyrrole NH group, thereby preventing competitive reactions at the amide site and the formation of regioisomers. This directing effect ensures that compounds **12** are produced with exclusive *trans*-stereoselectivity (d.r. > 20:1), as confirmed by ^1^H NMR analysis. This underscores the critical role of the OMP group in guiding the reaction pathway and maintaining high selectivity, even for the less reactive pyrrole derivatives.

**SCHEME 5:**
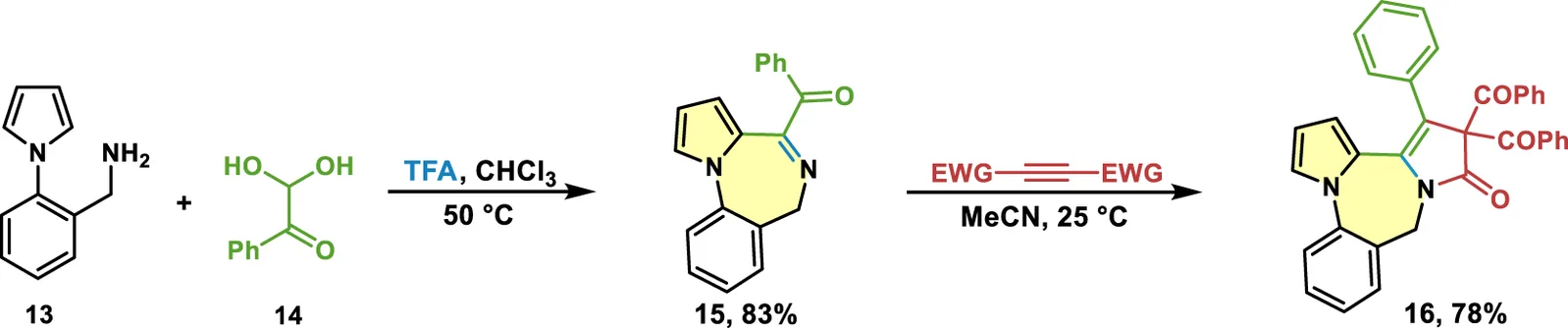
Synthesis of geminal dipyrrolo [1,2-a:2′,1′-c] [1,4]benzodiazepine 16 via Pictet-Spengler reaction and domino coupling with dibenzoylacetylene.

Building upon the versatile reactivity of pyrrolo[1,2-a][1,4]benzodiazepine cores, further structural modifications have facilitated the development of more intricate polycyclic frameworks. Notably, compound **16**, a geminal dipyrrolo[1,2-a:2′,1′-c][1,4]benzodiazepine derivative, was synthesized through a domino reaction between 4-aroylpyrrolo[1,2-a][1,4]benzodiazepine **15** and dibenzoylacetylene (DBA) under optimized conditions (Scheme 5) (Zinoveva et al., 2025). This reaction, conducted in acetonitrile at 25 °C for a duration of 2–3 h, resulted in an isolated yield of 78% for compound **16**. The precursor **15**, part of the 4-aroylpyrrolo[1,2-a][1,4]benzodiazepine (PBD) series, was synthesized via the Pictet-Spengler reaction. The key step in the formation of compounds **15** involves an intramolecular Pictet-Spengler-type cyclization, wherein the pyrrole ring attacks the iminium ion generated *in situ* from the condensation of the benzylamine moiety with the glyoxal derivative. This cyclization exhibits complete α-regioselectivity at the pyrrole C-2 position, furnishing the pyrrolo[1,2-*a*][1,4]benzodiazepine scaffold. This initial PBD served as a crucial starting material in the synthesis of dipyrrolo[1,2-a:2′,1′-c][1,4]benzodiazepine derivatives, providing the essential imino-ketone moiety necessary for the domino reaction with electron-deficient internal alkynes such as DBA.

**SCHEME 6:**
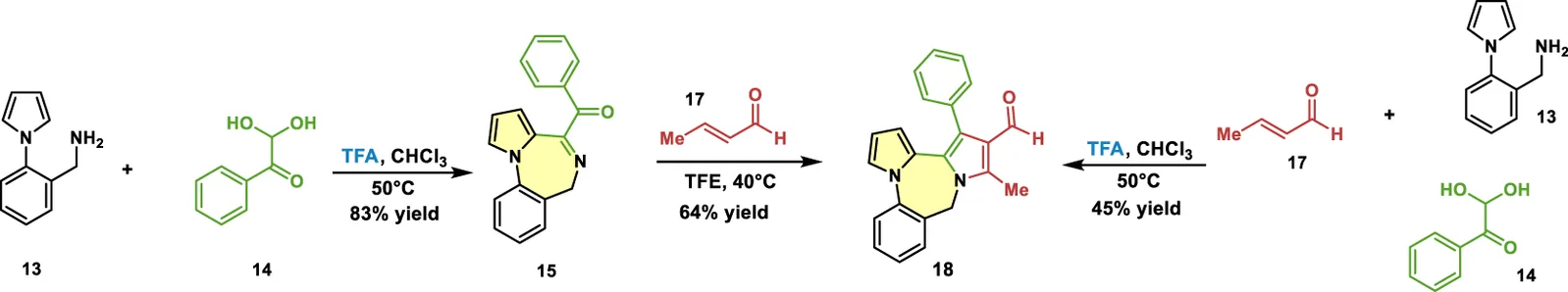
Synthesis of dipyrrolobenzo[1,2-a:2′,1′-c][1,4]diazepine scaffold via three-component reaction.

Heterocyclic compounds containing pyrrolo [1,4]benzodiazepine scaffolds have garnered significant attention due to their diverse biological activities, including analgesic, antifungal, and cytotoxic effects, as well as their interactions with the nervous system. The dipyrrolobenzo[1,2-a:2′,1′-c][1,4]diazepine scaffold, to which compound **18** is attributed, represents a novel structure in the realm of heterocyclic chemistry, thus rendering its synthesis particularly significant for the advancement of this field and the exploration of potential bioactive molecules (Scheme 6) (Zinoveva et al., 2024). The synthesis of compound **18** can be accomplished through two efficient methodologies, each emphasizing the formation of the critical 7-membered diazepine ring. In the domino reaction approach, compound **15** was initially synthesized from 1-(2-aminomethylphenyl)pyrrole **13** and arylglyoxal monohydrate **14** in chloroform, employing trifluoroacetic acid (TFA) as an acid catalyst at 50 °C. Subsequently, the PBD undergoes a domino reaction with electron-deficient alkenes **17** in TFA at 40 °C, culminating in the formation of the dipyrrolo[1,2-a:2′,1′-c][1,4]benzodiazepine derivative.

Alternatively, a one-pot three-component reaction (MCR) utilizing the same substrates directly constructs the scaffold. The reaction commenced with the sequential interactions of the amine **13** with arylglyoxal **14** and the alkene **17**, leading to the formation of an intermediate. This was followed by cyclization, accompanied by dehydration and dehydrogenation, ultimately yielding compound **18**, which retained the intact 7-membered diazepine ring, akin to that obtained via the domino approach. The efficient construction of this 7-membered ring from readily available precursors under mild conditions underscores the synthetic importance of compound **18**. Furthermore, its potent antimicrobial activity, with a minimum inhibitory concentration (MIC) of 8 μg/mL against *Staphylococcus aureus* ATCC-25923 and *Candida albicans* ATCC 10231, highlights its biological potential.

**SCHEME 7:**
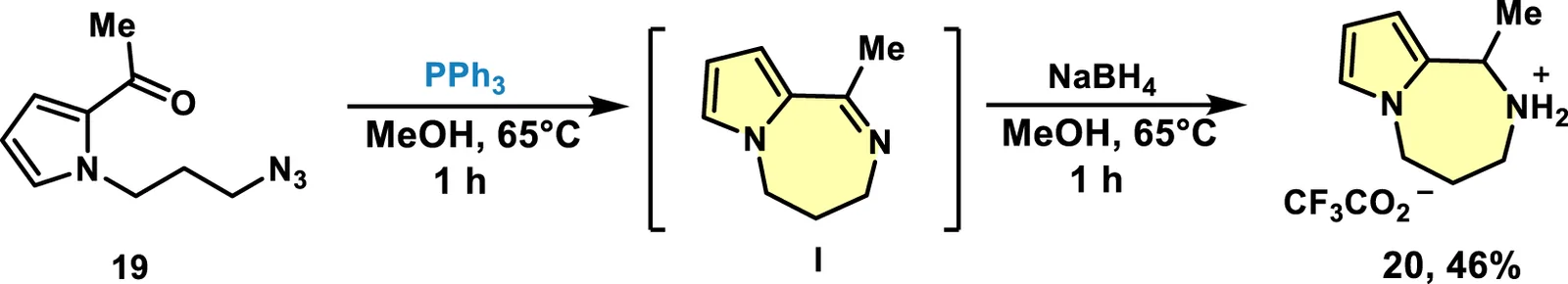
Synthesis of tetrahydropyrrolo [1,2-a]diazepine scaffold via intramolecular Staudinger/*aza*-Wittig tandem reaction followed by reduction.

In addition to annulation and domino strategies, intramolecular tandem reactions serve as a powerful method for the assembly of pyrrolo[1,2-a][1,4]diazepine cores with high efficiency and structural precision. The synthesis of compound **20** was achieved through an intramolecular Staudinger/aza-Wittig tandem reaction followed by reduction (Scheme 7) (Paramonova et al., 2024). Beginning with the corresponding azido-aldehyde **19**, the synthesis involved the use of PPh_3_ in anhydrous methanol, facilitating the Staudinger reaction of the azide group to form an iminophosphorane intermediate **I**. This intermediate subsequently underwent an *aza*-Wittig reaction, resulting in the elimination of triphenylphosphine oxide and the formation of an *in situ* imine. The imine was then reduced using NaBH_4_ under reflux conditions (65 °C, 1 h), yielding compound **20**, which was subsequently isolated as a trifluoroacetate salt through RP-HPLC purification. This methodology effectively constructed the seven-membered diazepine ring fused with a pyrrole moiety, demonstrating a high tolerance for various substituents and facilitating the synthesis of heterocyclic structures with medicinal relevance.

**SCHEME 8:**
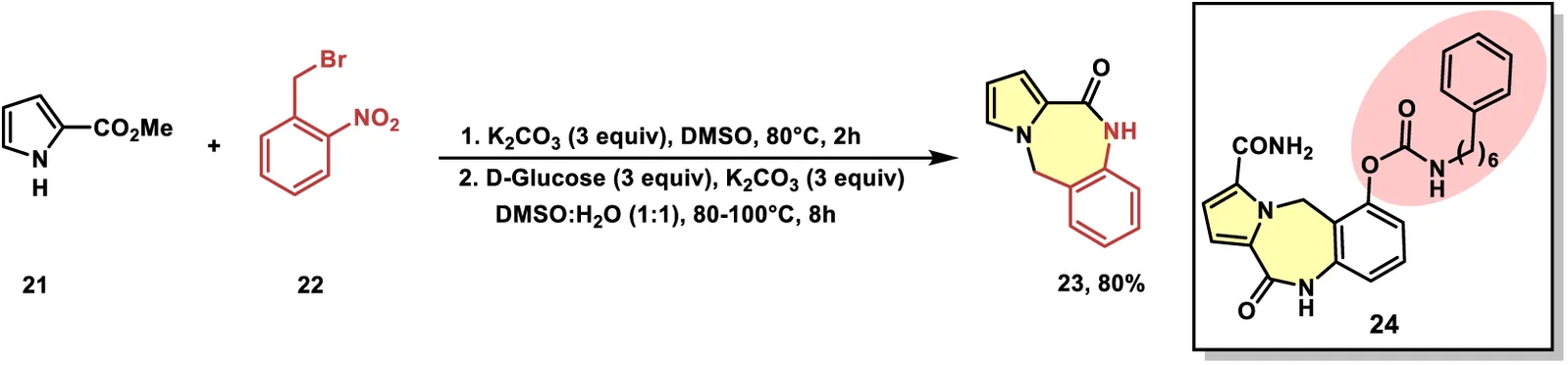
One-pot synthesis of pyrrolo[1,2-a][1,4]diazepin-11-one with via glucose-mediated nitro-reductive cyclization.

In addition to intramolecular tandem approaches, sustainable and biomolecule-assisted cyclization strategies have emerged as potent methodologies for the construction of bioactive pyrrolo[1,2-a][1,4]diazepine derivatives. Compound **24**, identified as pyrrolo[1,2-a][1,4]diazepin-11-one, was designed with potential anticonvulsant activity, aiming to broaden the biological applications of pyrrole-fused heterocycles in therapeutic chemistry (Scheme 8) (Panday et al., 2024). The synthesis of the target compound was accomplished through a one-pot glucose-mediated nitro-reductive cyclization process, utilizing 2-nitrobenzyl bromide **22** and methyl 1*H*-pyrrole-2-carboxylate **21** as substrates under standard reaction conditions. The reaction was specifically carried out in a DMSO:H_2_O (1:1) solvent system, employing D-glucose as a biomass-derived reducing agent and K_2_CO_3_ as the base. This procedure involved a sequential S_N_Ar reaction, reduction of the nitro group, and intramolecular cyclization, ultimately yielding compound **23** with an 80% yield.

This glucose-mediated one-pot tandem protocol exhibits excellent multi-layered chemo-, regio- and cyclization selectivity toward pyrrolo[1,2-a][1,4]diazepin-11-one, which is constructed from methyl pyrrole-2-carboxylate and 2-nitrobenzyl bromide without metal catalysts. First, exclusive S_N_2 *N*-benzylation occurs at the pyrrolic NH site under mild basic conditions. The aliphatic C–Br bond of 2-nitrobenzyl bromide acts as the sole electrophilic site. In contrast, the synthesis of six-membered pyrroloquinoxalines relies on 2-fluoronitroarenes with aromatic C–F bonds capable of S_N_Ar reaction. Since 2-nitrobenzyl bromide has no leaving groups on its aromatic ring, competitive S_N_Ar arylation pathways and dialkylation impurities are fully eliminated, delivering a single *N*-(2-nitrobenzyl) pyrrole carboxylate intermediate. Second, biomass-derived D-glucose displays high chemoselectivity for nitro group reduction: it selectively converts ortho-nitro substituents into primary amines via a two-electron transfer pathway, while ester carbonyls, benzyl linkages and aromatic backbones remain intact; no azo, hydrazine or carbonyl-reduction impurities are detected. Third, strict regioselective seven-membered lactam cyclization is governed by the preinstalled methylene bridge of the benzyl substrate.

**SCHEME 9:**
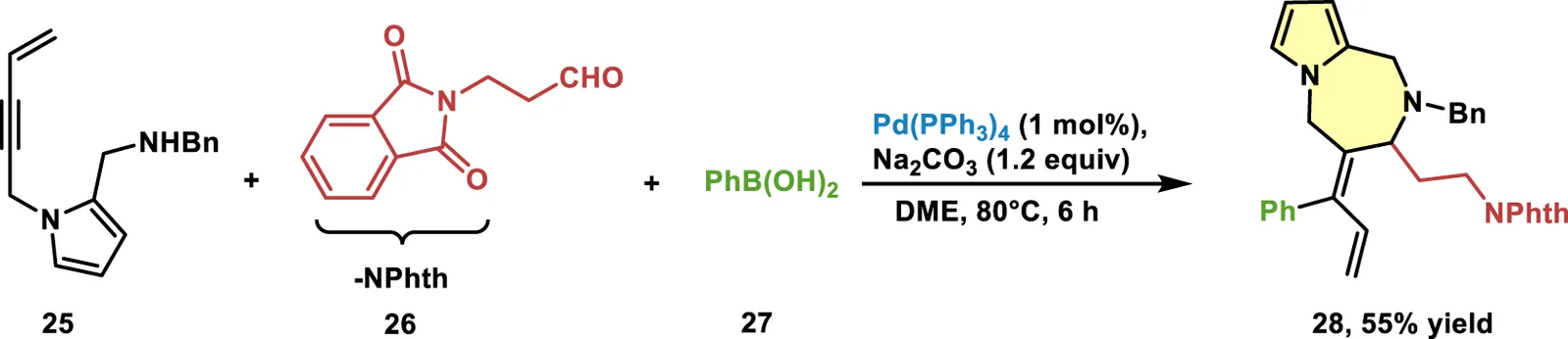
Palladium-catalyzed aminoalkylative cyclization for the synthesis of exocyclic 1,3-diene.

To further enhance the structural diversity of pyrrolo-fused seven-membered *N*-heterocycles, a transition-metal-catalyzed multicomponent cyclization strategy has been developed to access unique exocyclic diene architectures. Compound **28**, an exocyclic 1,3-diene featuring a seven-membered saturated *N*-heterocycle, was synthesized to expand the structural diversity of exocyclic diene derivatives containing medium-sized heterocycles (Scheme 9) (Zou et al., 2023b). This synthesis was achieved via a palladium-catalyzed regioselective aminoalkylative cyclization, a three-component reaction involving suitable aminoenynes **25** (with extended tether lengths to form seven-membered rings), aldehydes **26**, and boronic acids **27**. The reaction was carried out under mild conditions, utilizing 1 mol% Pd(PPh_3_)_4_ as the catalyst and Na_2_CO_3_ as the base in DME solvent at 80 °C for 6 h. This optimized protocol facilitated the selective formation of compound **28** with a yield of 55% and complete *E*-selectivity, demonstrating the versatility and precision of palladium-catalyzed aminoalkylative cyclization.

The aminoenyne precursor for compound **28** contains a four-methylene linker between the amine and enyne backbone, whose precise length establishes a spatial distance perfectly matching the geometric demands of selective seven-exo-trig cyclization; the substrate only bears a single enyne reactive motif and a simple *N*-benzyl group without extra pendant alkenes or alkynes, precluding competing intramolecular exo/endo cyclization pathways. This moderately flexible long alkyl tether readily folds to alleviate intrinsic transannular steric strain in the seven-membered ring transition state, whereas bulky substituents such as tert-butyl or polysubstituted aryl groups on the tether restrict conformational folding and drastically reduce the yield of seven-membered cyclic product.

The significance of compound **28** lies in its function as a versatile synthetic intermediate. Its conjugated diene moiety allows for participation in Diels-Alder reactions and cross-coupling reactions, thereby aiding in the construction of valuable spirocyclic systems, which are privileged motifs in natural products and pharmaceutically active molecules. Additionally, its seven-membered heterocyclic framework enriches the repertoire of *N*-heterocyclic compounds, offering potential scaffolds for drug discovery and further synthetic transformations.

**SCHEME 10:**
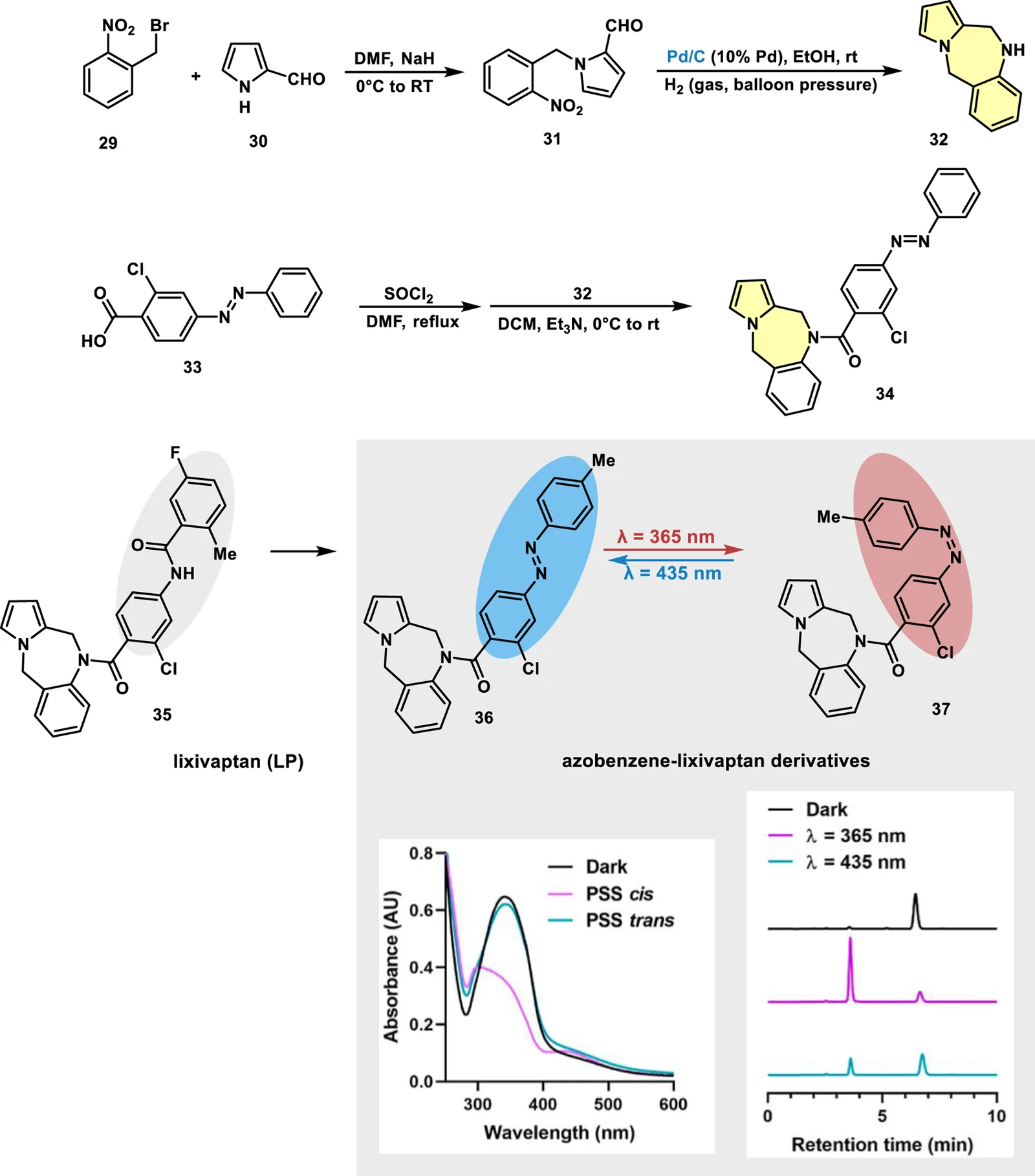
Synthesis and photochemical characterizations of photoswitchable compound 34 by integrating azobenzene into vasopressin V_2_R antagonist lixivaptan. Adapted with permission from Gu et al. (2023). Copyright 2023 American Chemical Society. RightsLink Order No. 6287640783364.

Beyond conventional synthetic targets, innovative strategies have been employed to construct functionalized pyrrolodiazepines with photoresponsive properties for precision medicine. The photoswitchable compound **34** was engineered to regulate the binding duration of a small-molecule ligand to its target receptor by incorporating an azobenzene moiety into the existing vasopressin V_2_ receptor (V_2_R) antagonist, lixivaptan (LP) (Scheme 10) (Gu et al., 2023). This modification was accomplished by substituting the amide group of LP with a bioisosteric azo fragment, thereby enabling optical control over its receptor binding kinetics. The synthesis of compound **34** involved several critical steps: initially, 2-nitrobenzyl bromide was reacted with pyrrole-2-carboxaldehyde to form intermediate **31**, which was subsequently reduced to yield intermediate **32**. Carboxylic acid **33** was then activated using SOCl_2_ and condensed with intermediate **32** to produce compound **34**.

This compound is capable of dynamically switching between its *cis* and *trans* isomers under 365 nm and 435 nm light irradiation. As demonstrated by the spectral results, the *trans* isomer dominates under dark conditions; 365 nm ultraviolet light induces the conversion to *cis* isomer, while 435 nm visible light drives the reaction back to the *trans* configuration. The HPLC results further quantify that the photostationary state contains 92% *cis* isomer under 365 nm irradiation, and 65% *trans* isomer after exposure to 435 nm light. The *cis* isomer demonstrates a prolonged residence time on the V_2_R (64 min) compared to the *trans* isomer (15 min). This dynamic control facilitates the fine-tuning of pharmacological activity, optimizing therapeutic efficacy while minimizing off-target side effects.

Furthermore, compound **34** introduces an innovative methodology in the field of photopharmacology, demonstrating the potential of utilizing light to dynamically modulate drug activity. Combined spectral and chromatographic data fully elaborate the rationality of its photopharmacological design. This approach can be extended to other G protein-coupled receptors (GPCRs) and drug targets, thereby offering novel avenues for the development of smart pharmaceuticals.

**SCHEME 11:**
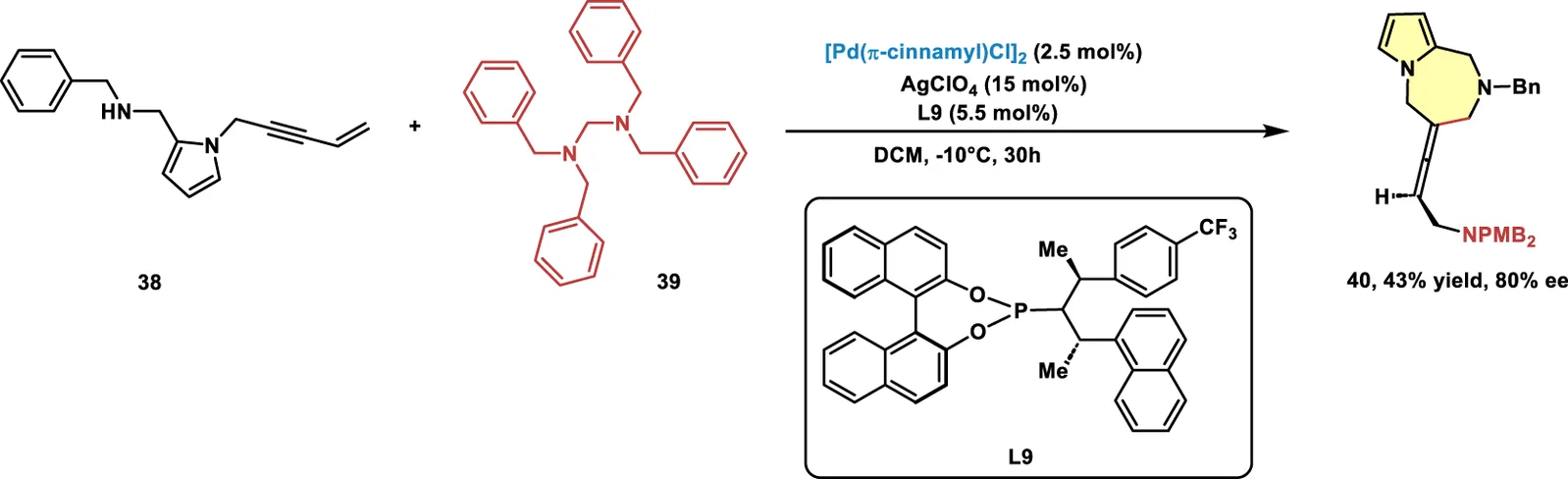
Palladium-catalyzed enantioselective ring-closing aminoalkylative amination for the synthesis of seven-membered *N*-heterocyclic exocyclic allenic amine 40.

For the construction of chiral pyrrolo-fused *N*-heterocycles, enantioselective palladium catalysis has enabled precise access to three-dimensional architectures with high stereocontrol. Compound **40** is a seven-membered *N*-heterocycle-containing exocyclic allenic amine, designed as a valuable synthon for constructing 3D heterocyclic architectures, which are privileged structures in drug discovery (Scheme 11) (Zou et al., 2023a). Its synthesis is achieved via a palladium-catalyzed enantioselective ring-closing aminoalkylative amination of unactivated aminoenynes **38**, leveraging a chiral phosphoramidite ligand (L9) for asymmetric induction.

The reaction proceeded with 2.5 mol% of [Pd (π-cinnamyl)Cl]_2_, 5.5 mol% of L9, and 15 mol% of AgClO_4_ as the catalytic system in DCM at −10 °C for 30 h. The synthesis process involved several key steps, beginning with the formation of a vinyl η^3^-allylpalladium intermediate through the intramolecular carbopalladation of the enyne. The adjacent amino group coordinated with palladium, thereby inhibiting undesired β-hydride elimination. A subsequent nucleophilic attack by the aminal at the less hindered electrophilic center of the η^3^-allylpalladium intermediate resulted in the formation of compound **40** with high enantioselectivity, underscoring its potential utility in synthesizing chiral saturated *N*-heterocycles pertinent to medicinal chemistry.

For the seven-membered ring product **40**, regioselectivity is governed by amino-directed intramolecular carbopalladation, wherein coordination of the tethered amine to palladium directs alkyne insertion while suppressing competing side reactions. Stereoselectivity is achieved through effective chiral induction by the phosphoramidite ligand **L9**, which facially discriminates nucleophilic attack at the η^3^-allylpalladium terminus. Despite the entropic penalty and transannular strain associated with medium-sized ring formation, the conformational rigidity imparted by the pyrrole moiety helps constrain the transition-state geometry, thereby preserving high enantiocontrol (80% ee) even in this otherwise disfavored seven-membered ring system.

**SCHEME 12:**
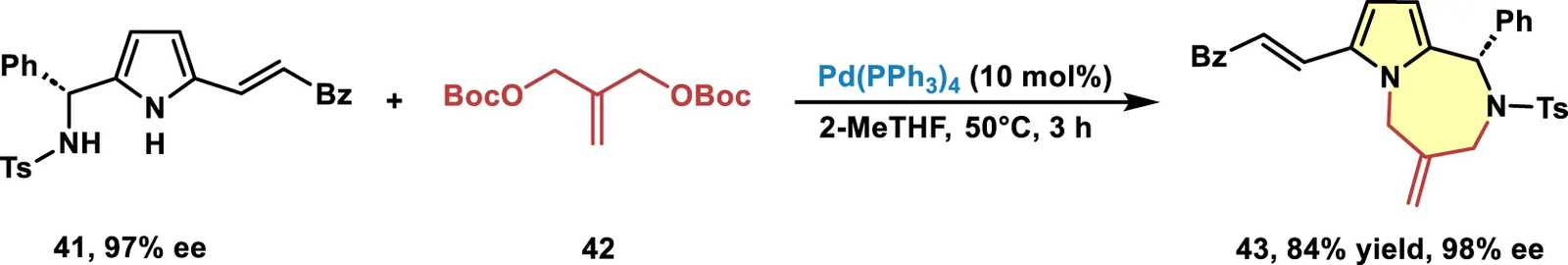
Palladium-catalyzed double *N*-allylation of pyrrole-derived FC adduct for the synthesis of diazepane 43.

Asymmetric C−C bond-forming reactions constitute a robust strategy for constructing complex pyrrolo-fused diazepane frameworks with precise stereochemical control. Compound **43** belongs to a series of complex, multifunctional adducts synthesized via an asymmetric Friedel-Crafts (FC) reaction (Scheme 12) (Jiang et al., 2023). The diazepane-fused pyrrole **43** was synthesized from the enantioenriched FC adduct **42**, which itself was obtained via a Pd^0^-catalyzed remote asymmetric FC reaction of a pyrrole substrate **41** bearing a two-enone tether. In this key C–C bond-forming event, the pyrrole C5-position undergoes enantioselective attack on an *N*-sulfonylimine electrophile under bifunctional phosphine ligand control, establishing the sole stereogenic center with high enantioselectivity. Leveraging the resultant allylic amine and pendant enone motifs within compound **41**, a subsequent palladium-catalyzed double *N*-allylation was executed. This intramolecular cascade sequentially forges two new C–N bonds, efficiently closing the seven-membered diazepane ring to afford the complex pyrrolo-fused framework **43** while faithfully preserving the stereochemical integrity set during the initial FC step.

The chemoselective double *N*-allylation of compound **41** to afford diazepane **43** relies on the differential reactivity of its two nitrogen nucleophiles: the free pyrrolic N–H undergoes initial allylation, while the tosyl-protected amino group requires palladium-mediated activation before the second *N*-allylation. The seven-membered ring formation is dictated by the allylic ester side chain length, which favors a seven-endo cyclization over alternative ring sizes such as the six-membered piperazine or the 20-membered macrocycle. Notably, the stereochemical integrity is fully preserved (97% ee to 98% ee) since the palladium-catalyzed process operates remotely from the existing stereogenic center. The use of Pd(PPh_3_)_4_ ensures π-Lewis basic rather than Lewis acidic catalysis, thereby suppressing side reactions at electron-deficient functionalities and securing high selectivity under mild conditions.

This transformation underscores the efficacy of the vinylogous strategy facilitated by Pd^0^ π-Lewis base catalysis, which not only ensures high efficiency in the formation of the desired heterocyclic structure but also preserves the stereochemical integrity derived from the chiral FC adduct. Consequently, compound **43** emerges as a valuable building block for further pharmaceutical and synthetic applications.

**SCHEME 13:**
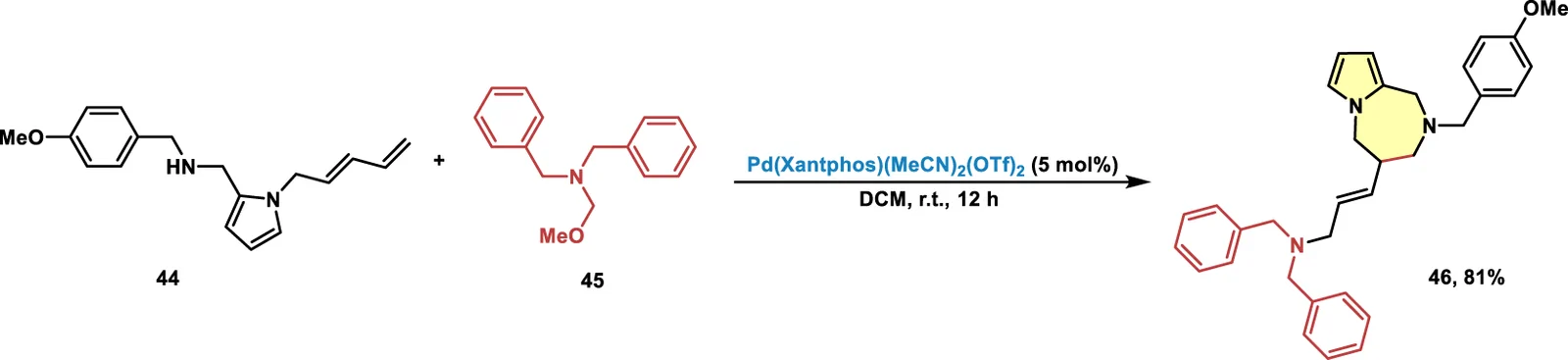
Palladium-catalyzed ring-closing reaction for the synthesis of compound 46 via C−N Bond metathesis.

Beyond asymmetric C−C bond formation, palladium-catalyzed C−N bond metathesis has gained prominence as an atom-economical approach to synthesizing saturated pyrrolo-fused *N*-heterocycles. The design of compound **46** focuses on constructing a saturated *N*-heterocyclic scaffold fused with a pyrrole ring, recognized as a privileged structure in medicinal chemistry due to its potential interactions with biological targets (Scheme 13) (Yu et al., 2021). The strategic incorporation of a 4-methoxybenzyl substituent and a dibenzylamino group is meticulously designed to enhance solubility, bioavailability, and binding affinity, while the preserved exocyclic allylamine functionality offers potential for further structural diversification.

Compound **46** was synthesized through a palladium-catalyzed ring-closing reaction. Under optimized conditions, the aminodiene substrate **44**, which features a pyrrole skeleton, was reacted with *N, O*-acetal **45** in the presence of 5 mol% Pd (Xantphos) (MeCN)_2_(OTf)_2_ as the catalyst in DCM at room temperature over a 12-h period. This reaction proceeded via C−N bond metathesis, involving the formation of a cyclopalladated complex from *N, O*-acetal, followed by intramolecular alkene migratory insertion and reductive elimination, ultimately yielding the target product with an 81% yield. The process demonstrates high atom economy, with methanol as the sole volatile byproduct, and the resulting fused heterocyclic structure serves as a valuable scaffold for drug discovery and natural product synthesis.

**SCHEME 14:**
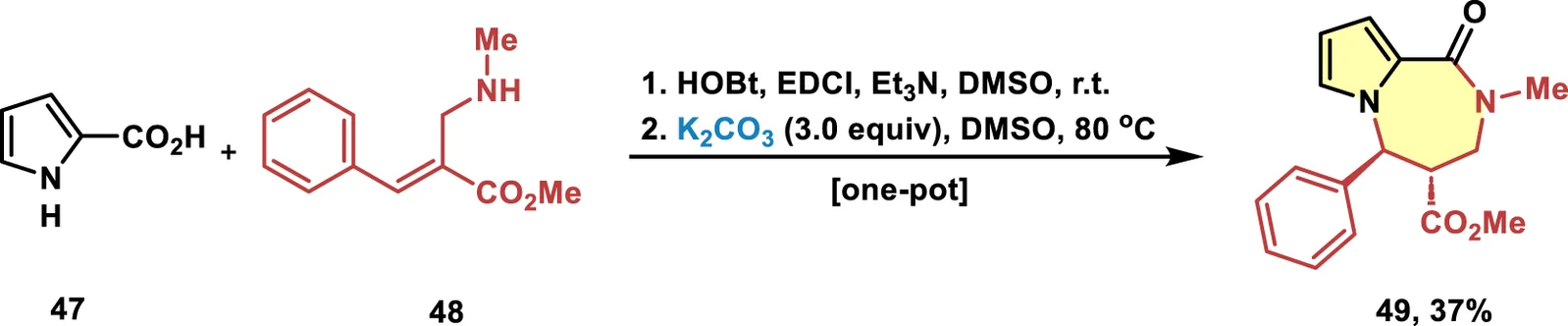
One-pot synthesis of compound 49 via sequential amide coupling/intramolecular *Aza*-Michael addition.

Modular, one-pot cyclization methodologies have been developed to facilitate the efficient synthesis of pyrrolo[1,2-a][1,4]diazepanones with precise stereochemical control (Scheme 14) (Wang et al., 2021). Notably, compound **49** exemplifies a pivotal pyrrole-fused 1,4-diazepanone framework, synthesized through a modular and stereoselective one-pot approach that incorporates sequential amide coupling followed by intramolecular *aza*-Michael addition. This compound was synthesized utilizing pyrrole-2-carboxylic acid as the pyrrole-containing precursor and a Morita-Baylis-Hillman (MBH)-derived allylamine (**48**, *E*-configured) as the diazepanone-forming synthon. Initially, 1*H*-pyrrole-2-carboxylic acid **47** was reacted with the MBH-derived allylamine **48** under standard coupling conditions to generate the linear amide intermediate *in situ*. The subsequent addition of K_2_CO_3_ and heating to 80 °C induced a seven-endo-trig cyclization, resulting in the formation of the target pyrrolo[1,2-a][1,4]diazepin-1-one **49** with a yield of 37%, exclusively as the *trans*-isomer.

The one-pot synthetic strategy for compound **49** exhibits excellent reaction selectivity control through three key aspects. Chemoselectivity is achieved via a temperature-programmed sequential process: amide coupling between pyrrole-2-carboxylic acid and the MBH-derived allylamine proceeds selectively at room temperature using HOBt/EDCI, while the intramolecular *aza*-Michael addition is triggered only upon heating to 80 °C with K_2_CO_3_, thereby preventing premature cyclization or competing side reactions; notably, the amide nitrogen serves as the exclusive nucleophile over the pyrrole N–H due to its enhanced nucleophilicity and the topological constraints of the tethered chain. Regioselectivity is governed by a highly favored seven-endo-trig cyclization pathway, which is thermodynamically and kinetically promoted by the three-atom linker geometry and the strongly polar DMSO solvent, representing the first reported intramolecular *aza*-Michael addition of pyrroles through this otherwise underdeveloped cyclization mode. Stereoselectivity is thermodynamically controlled under the basic reaction conditions (3.0 equiv K_2_CO_3_, 80 °C), where the *trans* configuration between the phenyl and ester substituents is exclusively favored as the more stable diastereomer, consistent with the indole-derived analogues.

**SCHEME 15:**
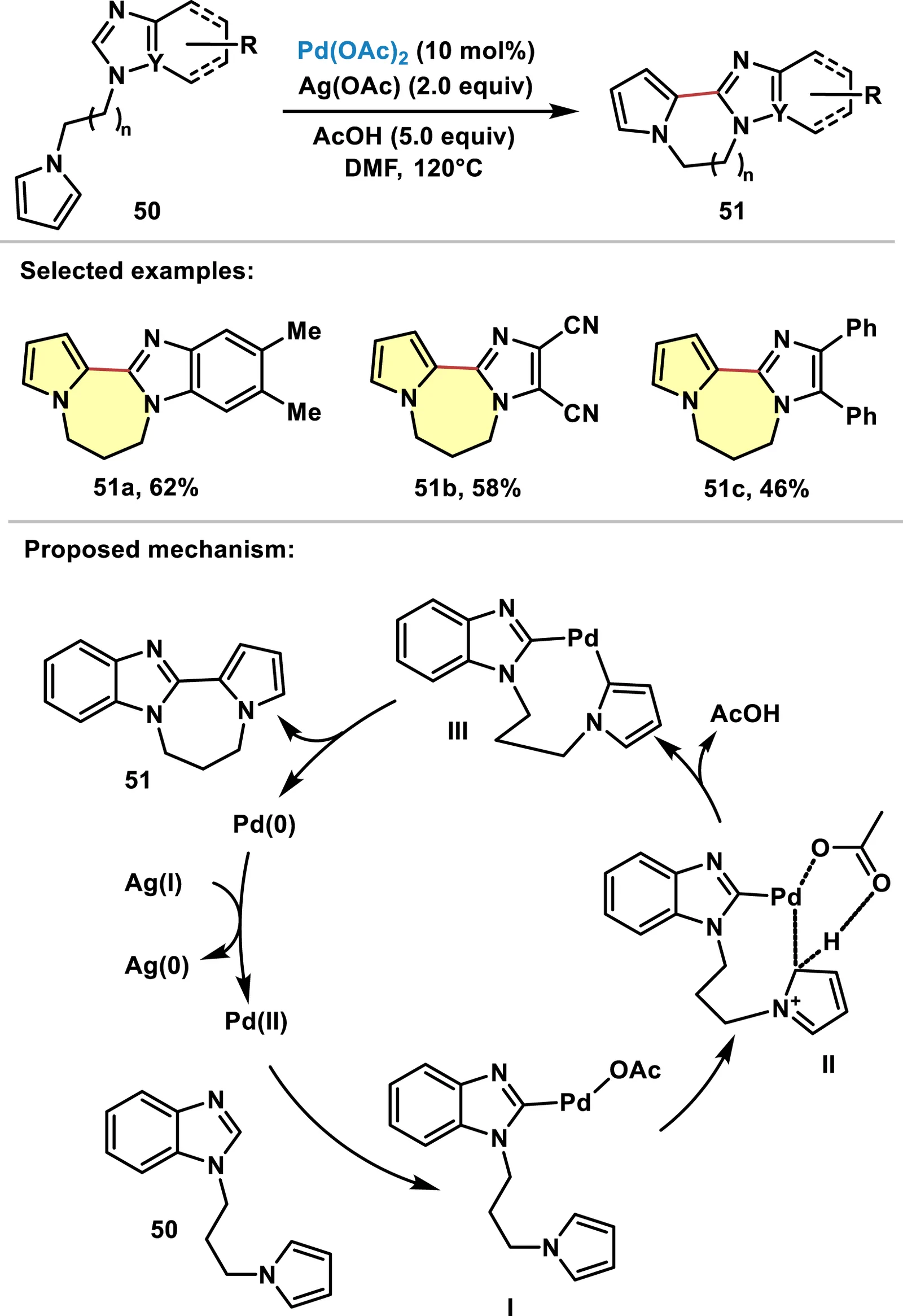
Synthesis and proposed mechanism for transformation of pyrrolylalkyl-*1H*-azoles to imidazopyrrolodiazepines via palladium-catalyzed intramolecular oxidative annulation.

Oxidative C−H/C−H coupling has emerged as a step-efficient strategy for synthesizing fused polyheterocyclic systems with pyrrolodiazepine cores. Tripathi et al. demonstrated a palladium-catalyzed intramolecular oxidative annulation method for the synthesis of imidazopyrrolodiazepines **51**, which are seven-membered ring polyheterocycles achieved by extending the alkyl chain linking pyrrole and azole moieties to three carbons (n = 2) (Scheme 15) (Tripathi et al., 2020). The pivotal cyclization was performed under palladium-catalyzed oxidative C–H/C–H conditions (10 mol% Pd(OAc)_2_, 2.0 equiv AgOAc, 5.0 equiv AcOH, DMF, 120 °C, 12 h), facilitating intramolecular dehydrogenative coupling between the imidazole C–2 and the pyrrole C–2, thereby forming the central diazepine ring with complete regioselectivity. This protocol accommodates electron-donating **51a,** electron-withdrawing **51b**, and extended π-systems **51c**, offering a dependable route to this class of polyheterocyclic arenes.

Compared with two-carbon-tethered six-membered products, three-carbon-linked seven-membered diazepine-fused polyheterocycles delivered substantially lower yields ranging from 46% to 71%, which verified that the elongated three-carbon alkyl linker increased conformational flexibility and disrupted the spatial alignment required for efficient double C–H bond activation, raising the energy barrier of concerted metalation-deprotonation and reductive elimination steps. Electron-donating methyl substituents caused larger yield losses than halide electron-withdrawing groups, and bulky 4,5-diphenyl-substituted gave the minimum yield (46%) due to aggravated steric repulsion.

The palladium-catalyzed dehydrogenative intramolecular oxidative coupling of pyrrolylalkyl-1*H*-azoles enables the selective construction of fused polyheterocyclic frameworks through a cascade involving dual C (sp^2^)–H bond functionalization. The observed regioselectivity, favoring initial C–H activation at the azole C2 position over the pyrrole counterpart, is consistent with the enhanced acidity of the former induced by proximal nitrogen atoms and was corroborated by intermolecular competition studies. The pronounced preference for intramolecular cyclization stems from the effective entropic and chelation-driven preorganization of the tethered substrate, which directs palladium coordination toward the proximal heteroarene and suppresses intermolecular pathways. Furthermore, the ring size of the annulated product can be modulated with high fidelity by varying the alkyl linker length, where two-atom tethers afford six-membered dihydropyrazine scaffolds and three-atom homologues furnish the corresponding seven-membered dihydrodiazepine architectures, underscoring the versatility of this annulation strategy.

##### 2.1.2.2 Pyrrolo[1,2-d][1,4]diazepine

**SCHEME 16:**
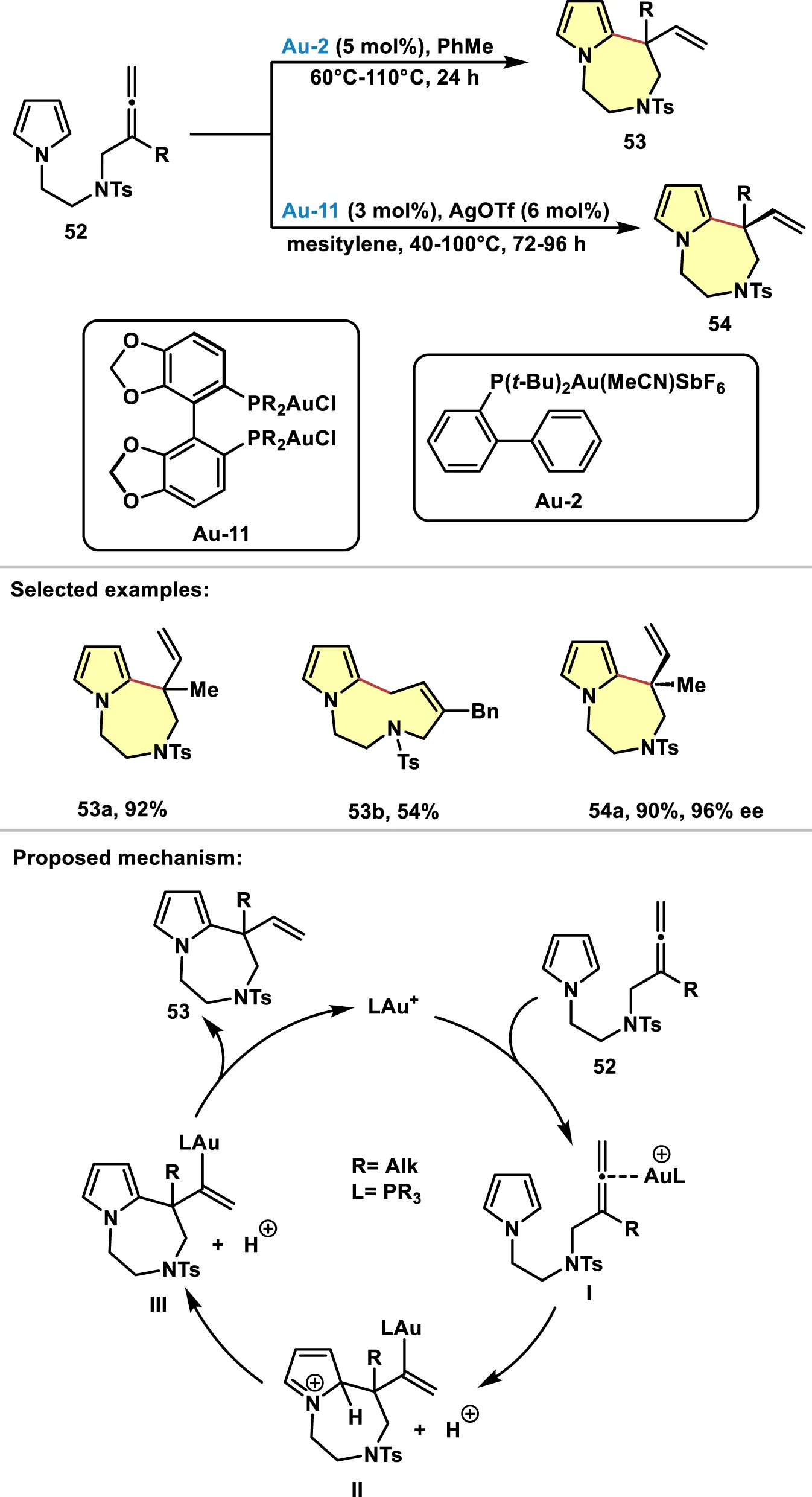
Synthesis and proposed mechanism for transformation of allenes to tetrahydrodiazepines via Au(I)-catalyzed regiodivergent hydroarylation.

Additionally, pyrrolo[1,2-d][1,4]diazepine, another significant regioisomer, has been efficiently synthesized using transition-metal-catalyzed strategies. The design of compound **53** focused on the synthesis of medium-sized nitrogen-containing heterocycles through regioselective Au(I)-catalyzed hydroarylation of pyrrole-tethered allenes, thereby addressing the synthetic challenge associated with the formation of seven-membered rings (Scheme 16) (Zhou et al., 2025). For the racemic synthesis of compound **53**, optimization efforts identified Au-2 as the most effective catalyst, facilitating seven-exo cyclization in toluene at temperatures ranging from 60 °C to 110 °C. Substrates possessing alkyl-gem-disubstituted allenes (such as methyl, ethyl, and *n*-butyl) yielded compound **53** with high efficiency (up to 92%) and excellent regioselectivity. In contrast, substrates with sterically demanding substituents (such as benzyl and cyclohexyl) exhibited a shift in selectivity towards the formation of nine-membered diazonines. The enantioselective synthesis of compound **54** was accomplished using (R)-DTBM-SEGPHOS as the chiral ligand and AgOTf as the counterion in mesitylene at 40 °C, resulting in enantioenriched compound **54a** with up to 96% enantiomeric excess and a 90% yield. The absolute configuration was confirmed as (S) via X-ray crystallography.

Mechanistic investigations and density functional theory (DFT) calculations indicate that the seven-exo cyclization is preferentially facilitated by the steric influences of alkyl substituents and the electron-donating properties of the JohnPhos ligand. These factors contribute to the stabilization of the gold-stabilized tertiary carbocation intermediate and guide the nucleophilic attack by the pyrrole moiety, followed by rearomatization and protodeauration, thereby regenerating the Au(I) catalyst.

**SCHEME 17:**
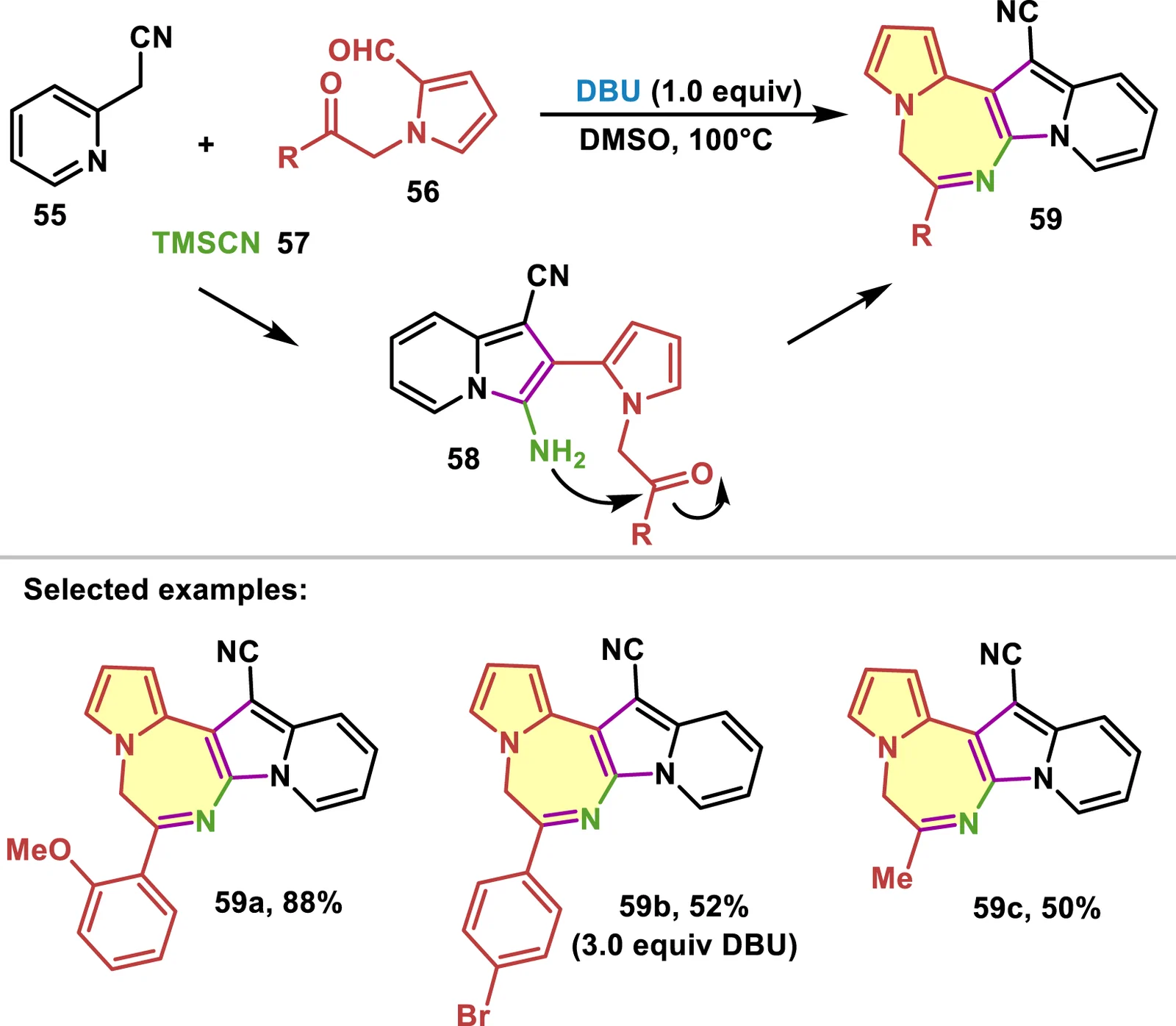
DBU-catalyzed one-pot three-component domino reaction for the synthesis of pyrrolodiazepine.

Beyond the realm of noble metal catalysis, organocatalytic multicomponent reactions present a milder and more cost-effective approach for the synthesis of pyrrolo[1,2-d][1,4]diazepine frameworks. The synthesis of pyrrolodiazepine **59** was accomplished through an efficient one-pot, three-component coupling reaction involving pyridine-2-acetonitrile **55**, *N*-substituted pyrrole-2-carboxaldehyde **56**, and TMSCN **57**, catalyzed by DBU in DMSO at 100 °C (Scheme 17) (Lee et al., 2025). This reaction proceeds via a domino sequence encompassing aldol condensation, Michael addition, cycloisomerization, and intramolecular imine formation, facilitating the sequential construction of the pyrrole and diazepine rings with the formation of two C–C and two C–N bonds.

The optimization of the reaction conditions revealed that utilizing 1 equivalent of DBU and DMSO as the solvent was essential for achieving high yields. In contrast, alternative bases such as DBN, K_2_CO_3_, and TEA, as well as different solvents like EtOH, MeCN, and THF, resulted in diminished efficiency or no reaction. The evaluation of substrate scope indicated that *N*-substituted pyrrole-2-carboxaldehydes with electron-rich aryl groups produced compound **59** in good yields. However, substrates with halogen-substituted aryl groups necessitated an increased DBU loading of 3 equivalents to enhance yields.

**SCHEME 18:**
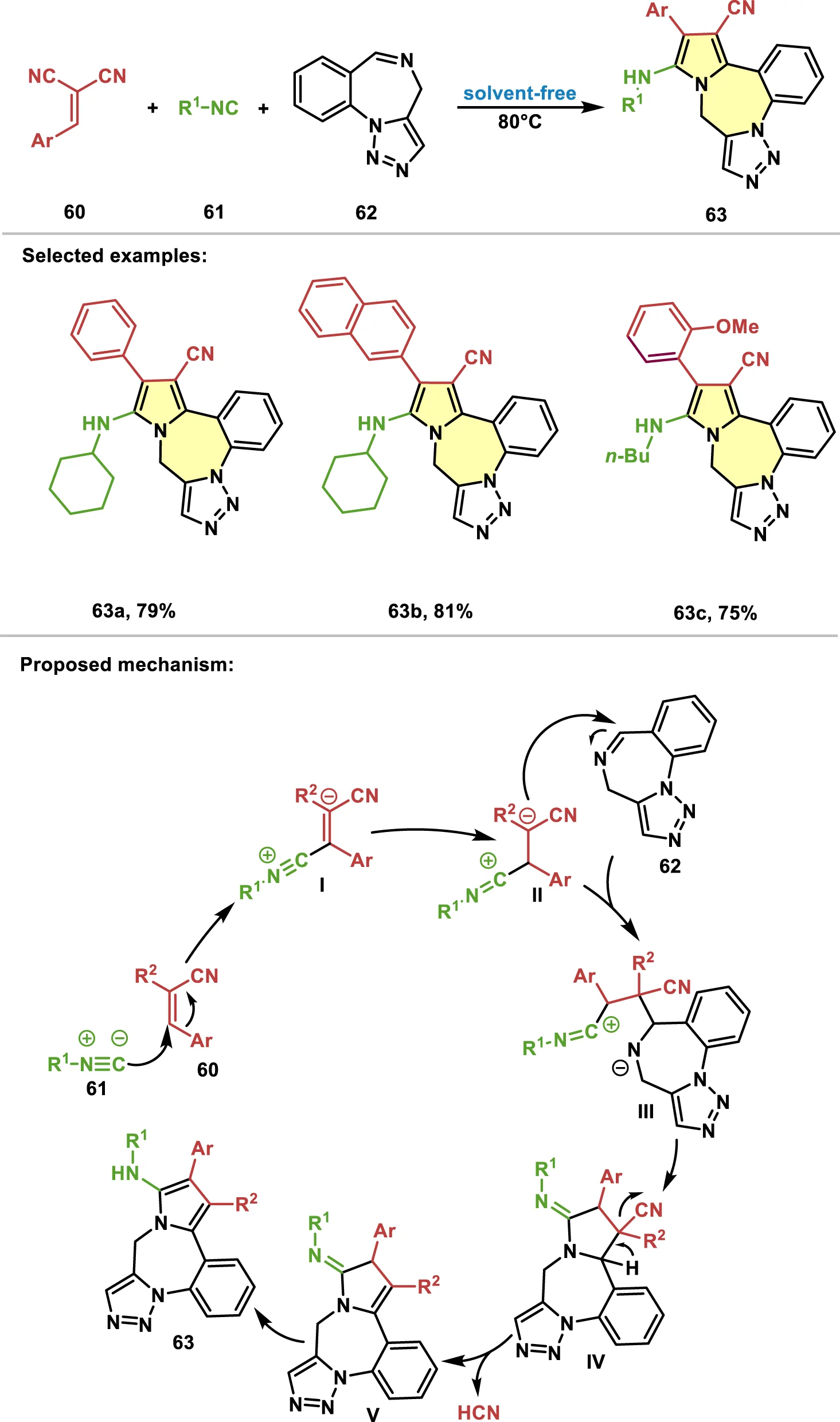
Synthesis and proposed mechanism of pyrrole-fused triazolobenzodiazepine derivatives via solvent- and catalyst-free isocyanide-based multicomponent reactions.

To enhance the structural diversity of pyrrole-fused diazepines, isocyanide-based multicomponent reactions (I-MCRs) have been innovatively developed under conditions devoid of solvents and catalysts. A highly efficient and straightforward synthetic pathway for the generation of pyrrole-fused triazolobenzodiazepine **63** has been established through I-MCRs involving cyclic imines **62**, geminally di-activated olefins **60**, and isocyanides **61**, also under solvent- and catalyst-free conditions (Scheme 18) (Norouzi et al., 2024). The optimal reaction conditions were identified as stirring the reactants at 80 °C for 2 h, yielding the desired products with high efficiency, ranging from 72% to 91%. A broad range of gem-diactivated olefins with electron-donating (–Me, –OMe) and halogen (–Cl, –Br) substituents on the aromatic ring were well tolerated, and the presence of electron-withdrawing groups slightly enhanced the reaction yield; naphthyl-substituted gem-diactivated olefins also showed higher reactivity compared to phenyl-substituted counterparts. Various isocyanides (e.g., cyclohexyl, *n*-butyl isocyanide) were compatible with the protocol, enabling structural diversification of the products.

The mechanism proceeds via nucleophilic attack of the isocyanide on the olefin to generate a zwitterionic intermediate, which undergoes addition to the cyclic imine, cyclization, elimination of HCN and final enamine–imine tautomerisation to construct the pyrrole-triazolobenzodiazepine core.

**SCHEME 19:**
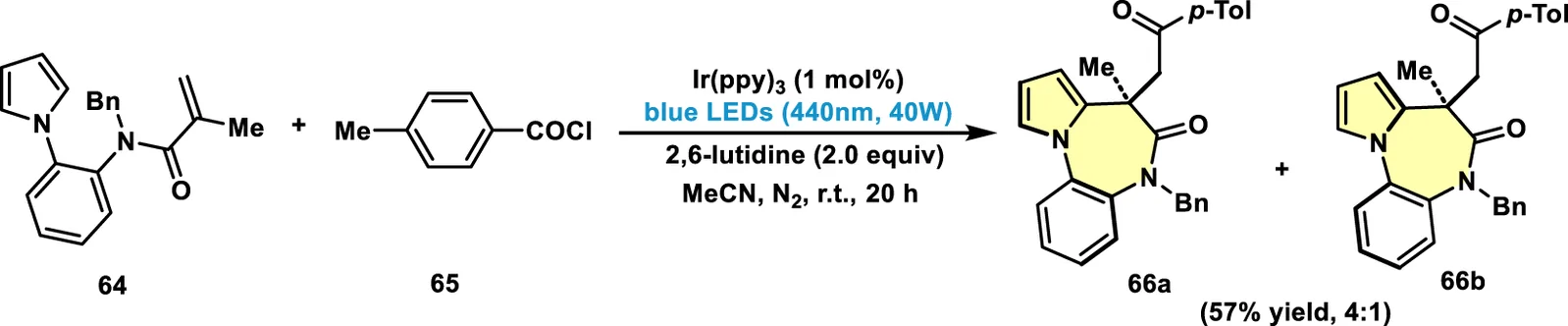
Photoredox-catalyzed cascade cyclization for the synthesis of pyrrole-fused 1,4-diazepinones.

In addition to conventional ionic and polar cyclizations, visible-light photoredox catalysis has emerged as a mild and environmentally friendly approach for the synthesis of pyrrole-fused 1,4-diazepinones. Brambilla et al. have documented the synthesis of compound **66** through a photoredox-catalyzed cascade cyclization reaction, utilizing compound **64** and aroyl chloride **65** as the radical source (Scheme 19) (Brambilla et al., 2023). Under optimized conditions (1 mol% Ir (ppy)_3_ as the photocatalyst, 2,6-lutidine as the base, and MeCN as the solvent, irradiated with 40 W blue LEDs at room temperature for 20 h), the reaction proceeded through sequential acyl radical addition to the C=C double bond of the methacrylamide moiety and intramolecular cyclization at the C2-position of the pyrrole ring. This cascade process yielded compound **66** as a pair of diastereomers in a satisfactory 57% yield with a diastereomeric ratio of 4:1.

The amide group is an indispensable chemoselective determinant, without which the cascade fails entirely; the complete loss of reactivity upon ester substitution reveals that this carbonyl operates not merely as a passive linker but as an active participant in stabilizing key radical intermediates or directing the ring closure. Meanwhile, the pyrrole scaffold, though structurally concise, furnishes a distinct steric and electronic microenvironment that facilitates precise selectivity control in these photoredox-catalyzed annulations.

**SCHEME 20:**
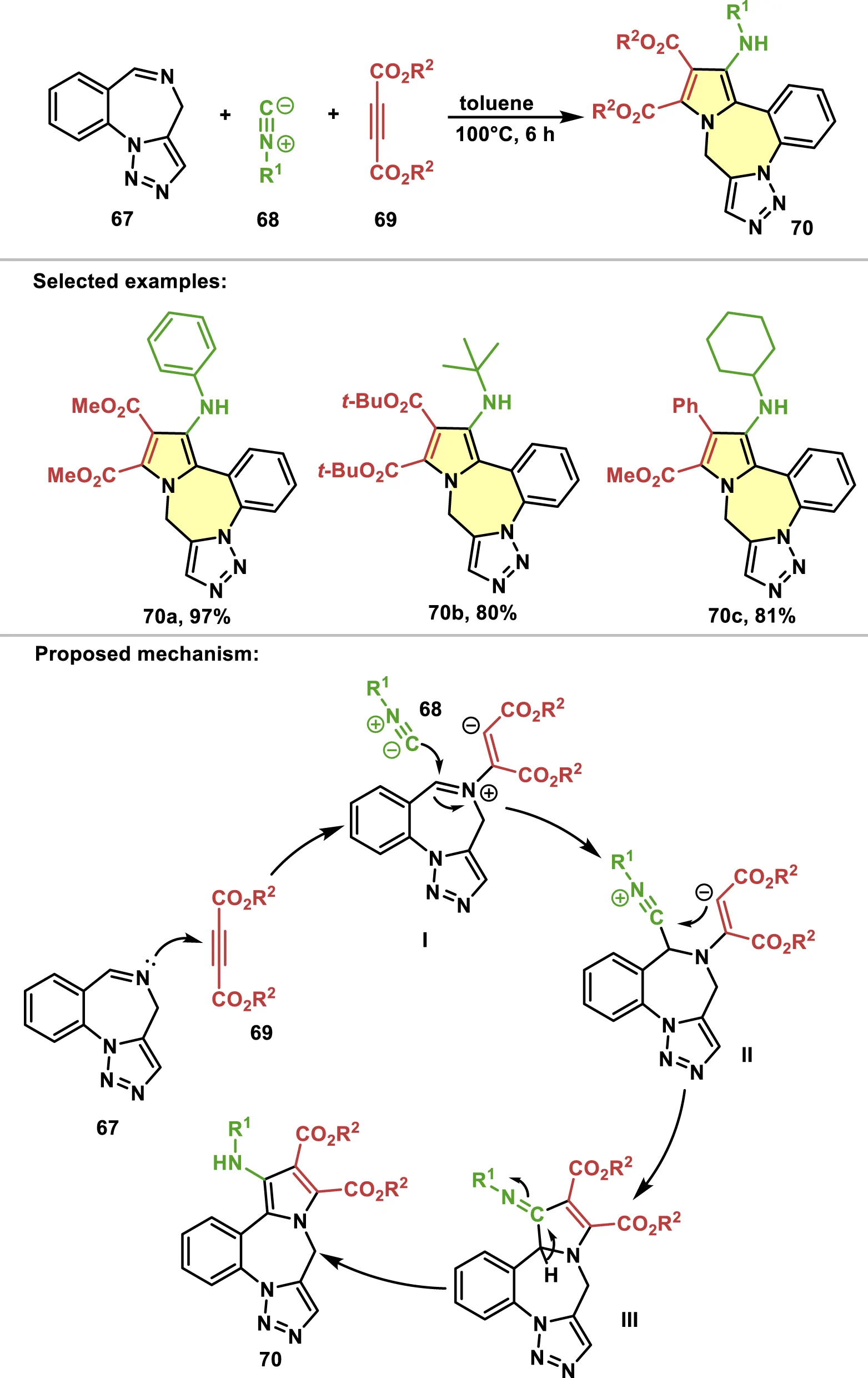
Catalyst-free Pseudo-Joullié−Ugi three-component reaction for the synthesis of triazolobenzodiazepine-fused pyrroles and proposed mechanism.

Catalyst-free multicomponent reactions offer a sustainable method for the efficient construction of triazolobenzodiazepine-fused pyrrole structures. The synthesis of these fused pyrroles was accomplished through a catalyst-free pseudo-Joullié−Ugi three-component reaction, utilizing triazolobenzodiazepine imine **67** as the cyclic imine, alongside isocyanides **68** and acetylenedicarboxylates **69** as principal substrates (Scheme 20) (Nazeri et al., 2023). The optimized reaction conditions included the use of toluene as the solvent, a temperature of 100 °C, and a duration of 6 h, resulting in the production of the desired compounds **70** with high yields, reaching up to 97%, and excellent chemoselectivity. The reaction demonstrated a broad substrate scope, accommodating various isocyanides and acetylenedicarboxylates, with the steric and electronic properties of substituents exerting a minor influence on yields without affecting chemoselectivity.

Mechanistically, the reaction proceeds via the initial formation of a 1,4-dipole zwitterion between the triazolobenzodiazepine imine **67** and acetylenedicarboxylate **69**, which is subsequently intercepted by the isocyanide **68**, followed by intramolecular cyclization and a [1,3]-H shift to produce the fused pyrrole framework. Significantly, this catalyst-free pseudo-Joullié–Ugi reaction proceeds exclusively via a 1,4-dipole zwitterion pathway and fully blocks the competing side route common to isocyanide/acetylenedicarboxylate systems. Unlike conventional systems where isocyanides first combine with alkynyl esters to form undesired pyrrole precursors, the nucleophilic C=N bond of triazolobenzodiazepine imines preferentially attacks alkynyl electrophiles to generate imine-based 1,4-dipoles, which are subsequently trapped by isocyanides. Single-crystal XRD confirms sole formation of fused pyrroles with no cross-pathway byproducts, proving excellent chemoselectivity. The synthesized compounds demonstrate distinct photophysical properties, characterized by absorption in the range of 225–400 nm and emission between 400 and 560 nm, thereby positioning them as promising candidates for optoelectronic materials.

**SCHEME 21:**
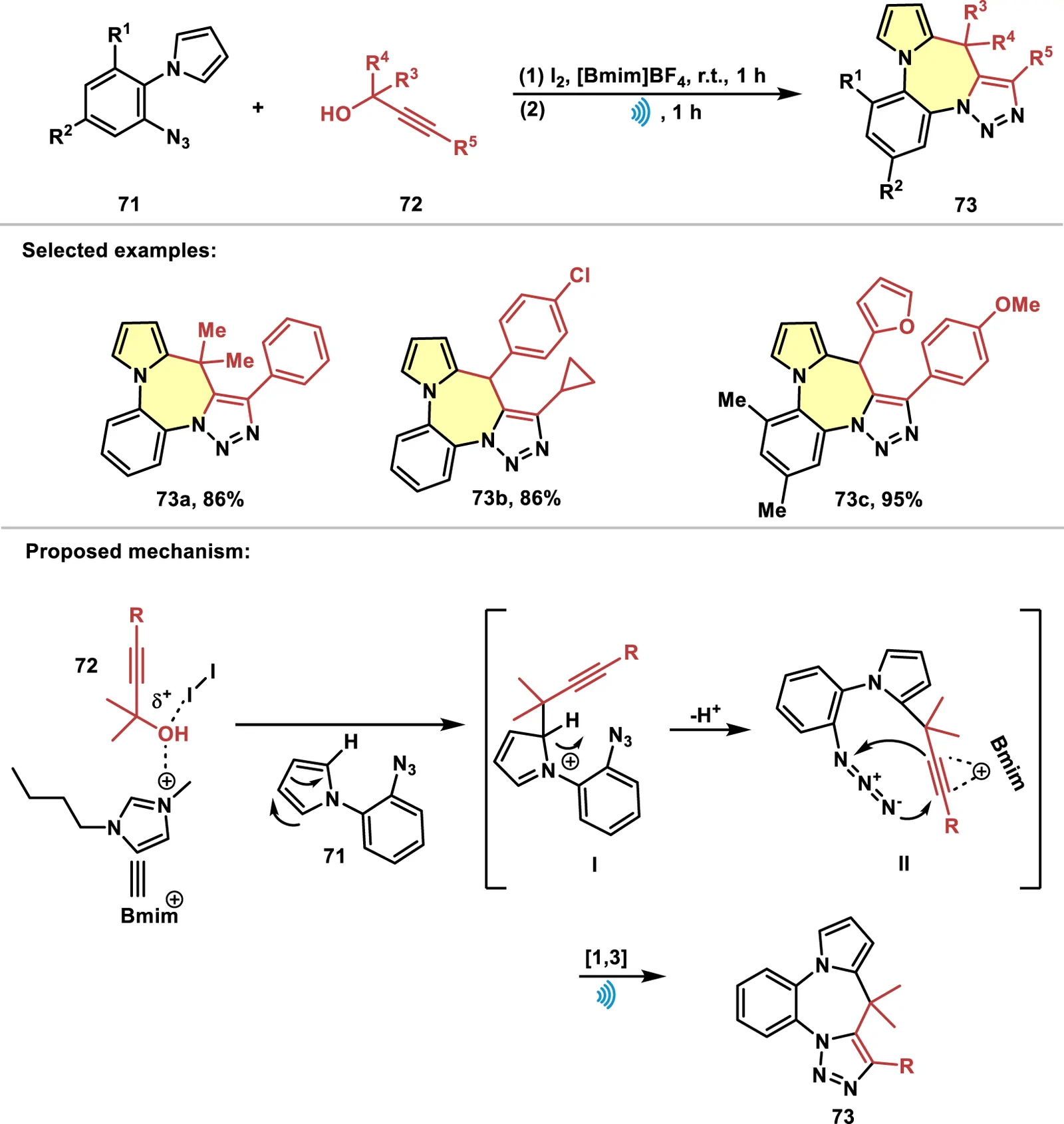
Synthesis and proposed mechanism of pyrrolobenzodiazepine-triazole scaffold via ultrasound-assisted ionic liquid-molecular iodine synergistic domino reaction.

Furthermore, green synthetic methodologies, facilitated by ultrasound and ionic liquids, have been employed to construct pyrrolobenzodiazepine-triazole hybrid scaffolds (Scheme 21). The synthesis of pyrrolobenzodiazepine-triazole hybrids **73** was achieved through an efficient, environmentally friendly domino protocol, utilizing ultrasound assistance and a synergistic catalytic system comprising molecular iodine and the ionic liquid [Bmim]BF_4_ (Saquib et al., 2023). In this process, the precursors 1-(2-azidoaryl)-1*H*-pyrroles **71** and propargyl alcohols **72** underwent sequential intermolecular electrophilic substitution at the C–2 position of the pyrrole ring, followed by intramolecular Huisgen 1,3-dipolar azide-alkyne cycloaddition. The optimized reaction conditions, involving 10 mol% I_2_, [Bmim]BF_4_ as the solvent/reaction medium, a temperature of 40 °C, and ultrasonic irradiation, resulted in high yields of up to 90%, with operational simplicity, a broad substrate scope, and the recyclability of the ionic liquid for up to four cycles with minimal yield loss.

The reaction mechanism consists of two principal sequential steps. Initially, molecular iodine and [Bmim]^+^ interact with the oxygen atom of propargyl alcohol, leading to its activation. This activation facilitates an electrophilic substitution reaction at the C–2 position, identified as the most nucleophilic site, of the pyrrole precursor, resulting in the formation of a C–2 propargylated pyrrole intermediate **I** accompanied by re-aromatization. Subsequently, ultrasonic irradiation induces the intramolecular Huisgen 1,3-dipolar azide-alkyne cycloaddition between the azide group of the aryl moiety and the alkyne group of the propargylated intermediate **II**. This process culminates in the construction of the fused pyrrolobenzodiazepine-triazole scaffold.

The exceptional selectivity and high yield observed for compound **73** arise from a synergistic interplay of precisely tuned reaction parameters. The catalytic system comprising 10 mol% molecular iodine and [Bmim]BF_4_ activates the propargyl alcohol via Lewis acid–base interactions while avoiding over-activation that would trigger competing side reactions. Regioselective C–2 propargylation of the pyrrole nucleus is ensured through a temperature-programmed protocol, with subsequent ultrasound-promoted intramolecular Huisgen 1,3-dipolar cycloaddition effectively suppressing intermolecular pathways and substrate decomposition. The strictly metal-free conditions favor thermodynamically controlled formation of the 1,5-disubstituted 1,2,3-triazole regioisomer, distinct from the 1,4-regioselectivity typically enforced by Cu(I)-catalyzed azide–alkyne cycloaddition. Furthermore, the ionic liquid medium creates a polar yet non-nucleophilic microenvironment that stabilizes ionic intermediates, with its inherent viscosity attenuating diffusion-controlled side reactions. Substrate design also plays a critical role: the gem-dimethyl group exerts a Thorpe–Ingold effect that accelerates cyclization, while the electron-donating R^5^ substituent stabilizes the alkyne π-system, collectively ensuring efficient and selective construction of the pyrrolobenzodiazepine-triazole hybrid scaffold.

**SCHEME 22:**
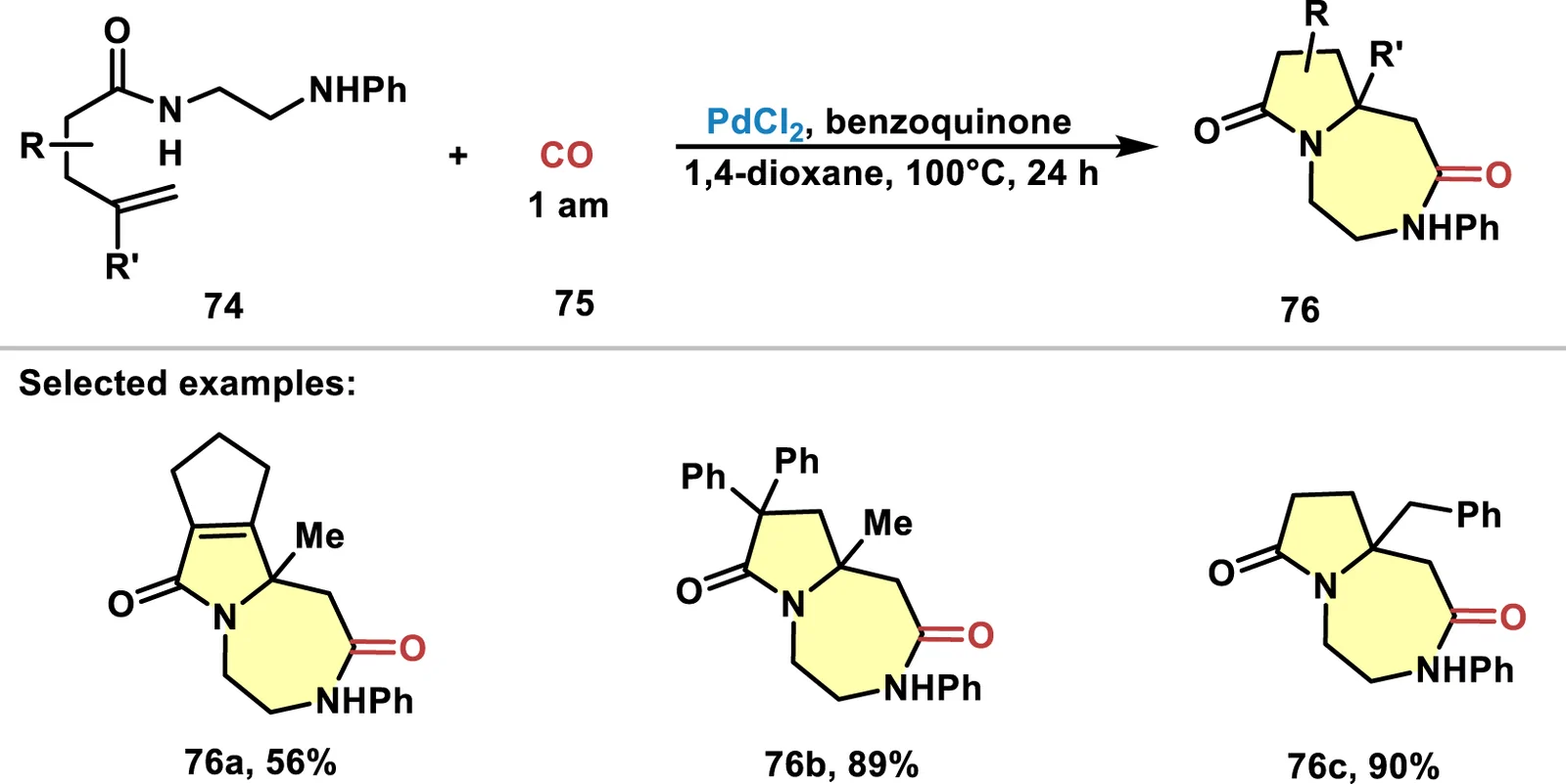
Palladium-catalyzed tandem carbonylative aza-Wacker-type cyclization of pyrrole-tethered alkenylamides for the synthesis of fused 1,4-diazepanones.

Finally, palladium-catalyzed carbonylative cyclization offers a powerful strategy for the construction of carbonyl-containing pyrrole-fused 1,4-diazepanones. Compound **76** referred to a series of pyrrole-fused 1,4-diazepanones, which were constructed via a palladium-catalyzed tandem carbonylative aza-Wacker-type cyclization (Scheme 22) (Shi et al., 2021). The molecular design strategy focused on nucleophile-tethered alkenylamide frameworks. The synthesis was conducted under optimized conditions, employing 1 mol% PdCl_2_ as the catalyst, benzoquinone as the oxidant, 1,4-dioxane as the solvent, and 1 atm of CO at 100 °C for 24 h. To ensure compatibility with the catalytic system, the pyrrole moiety or pyrrole-fused framework was strategically integrated into the substrate structure. Concurrently, structural diversification of the alkenylamide component **74** was undertaken to systematically explore the substrate scope and assess the catalytic system’s adaptability.

This protocol effectively produced the desired pyrrole-fused 1,4-diazepanones, with yields varying from moderate to excellent. Specifically, compound **76a** was obtained in a moderate yield, likely due to the ring strain associated with cyclic alkenes. In contrast, compounds **76b** and **76c** were synthesized with excellent yields, with compound **76c** achieving a yield of 90%.

### 2.2 Methods for the synthesis of 1,3-diazepines fused pyrrole

In contrast to the well-established synthetic strategies for 1,4-diazepine-fused pyrrole systems, pyrrole-fused 1,3-diazepines have emerged as underexplored yet attractive heterocyclic scaffolds with unique structural characteristics. Within this category, N–C fused pyrrolo [1,3]diazepines constitute a major structural subclass, and their representative synthetic methods are detailed in the following section.

#### 2.2.1 N–C fused

Among the N–C fused pyrrolo [1,3]diazepine frameworks, two representative regioisomeric scaffolds have been successfully constructed via modern synthetic strategies: pyrrolo[1,2-c][1,3]diazepine and pyrrolo[1,2-a][1,3]diazepine. These two subtypes exhibit distinct ring-fusion topologies and ring-closure patterns, and their synthetic approaches are introduced sequentially in the following subsections.

##### 2.2.1.1 Pyrrolo[1,2-c][1,3]diazepine

**SCHEME 23:**
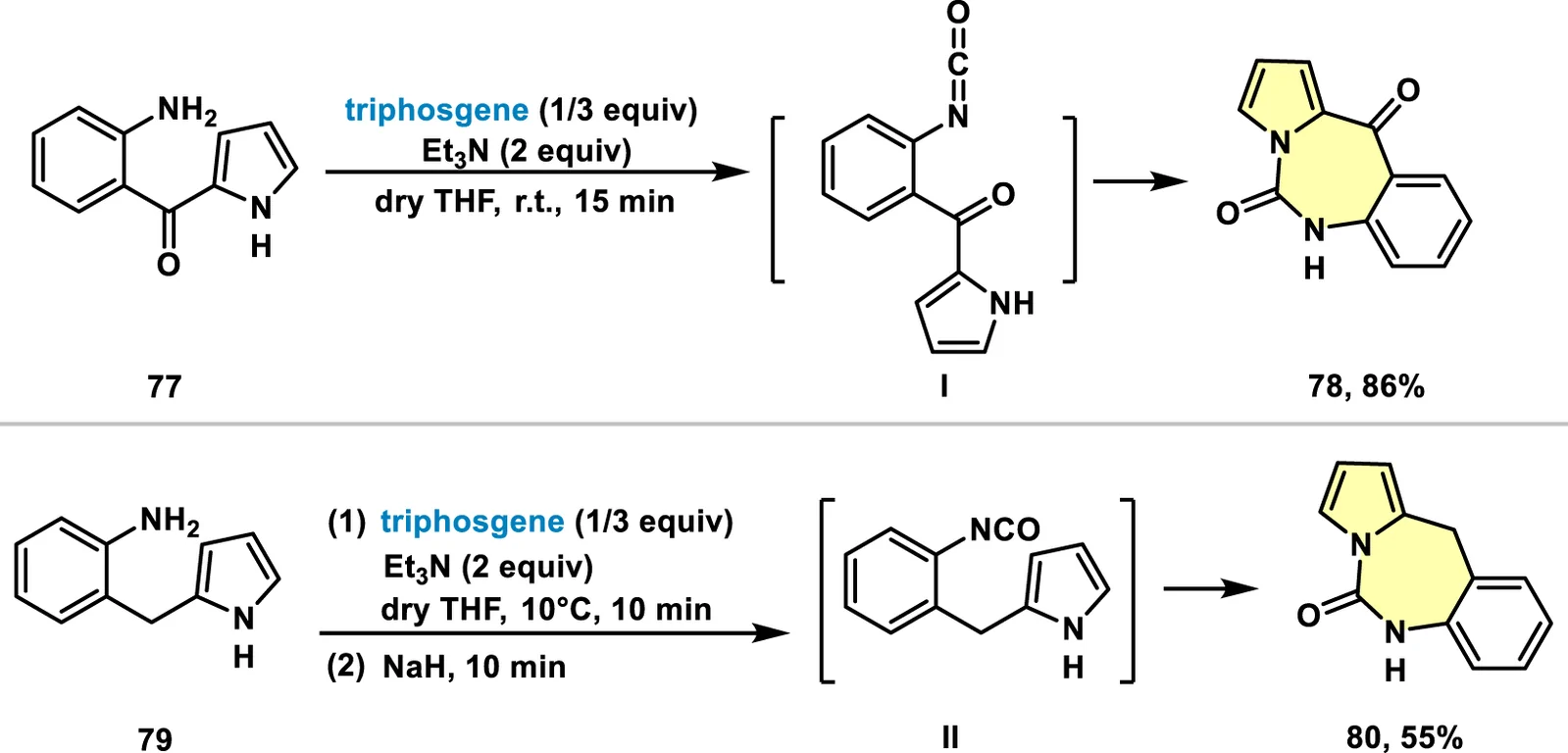
Synthesis of pyrrole-based tricyclics via triphosgene-Et_3_N System.

Rotas and Varvounis reported the synthesis of two novel pyrrole-based tricyclic heterocycles, utilizing triphosgene as the carbonylation-cyclization reagent to construct the seven-membered heterocycle (Scheme 23) (Rotas and Varvounis, 2020). Compound **78** was synthesized via triphosgene-mediated carbonylation-cyclization of (2-aminophenyl) (1*H*-pyrrol-2-yl)methanone **77**. The reaction involved treating amine **77** with one-third equivalent of triphosgene and two equivalents of Et_3_N in anhydrous THF at ambient temperature for 15 min. Subsequent aqueous workup with saturated NaHCO_3_ facilitated the cyclization of the unstable isocyanate intermediate **I**, resulting in the formation of compound **78** as white crystals with an 86% yield.

For the synthesis of compound **80**, the precursor 2-[(1*H*-pyrrol-2-yl)methyl]aniline **79** was utilized. The absence of an electron-withdrawing keto group in compound **79** necessitated alterations in the reaction conditions, which included one-third equivalent of triphosgene, two equivalents of Et_3_N and NaH. NaH was employed to deprotonate the isocyanate intermediate **II** and prevent hydrolytic ring-opening. A meticulously controlled workup procedure was implemented to isolate compound **80** with a 55% yield, involving sequential air stirring, dilution with DCM, and final quenching.

##### 2.2.1.2 Pyrrolo[1,2-a][1,3]diazepine

**SCHEME 24:**
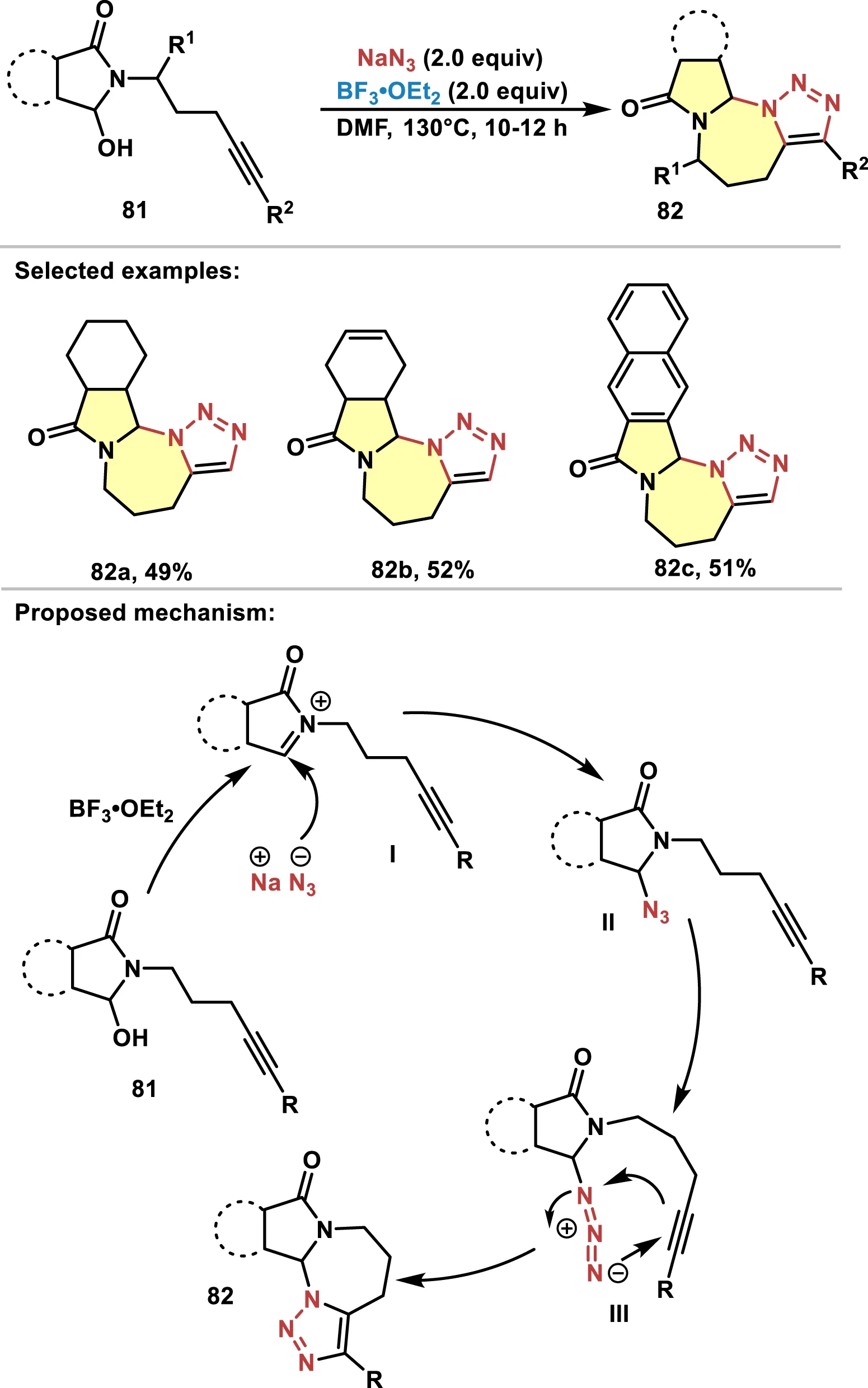
Synthesis and proposed mechanism of triazolo-diazepinoisoindolones via BF_3_·OEt_2_-mediated intramolecular Huisgen cycloaddition.

The transition-metal-free Huisgen cycloaddition presents a versatile methodology for synthesizing triazole-fused pyrrolo[2,1-b][1,3]diazepine frameworks. This innovative class of triazole-fused *N*-heterocycles **82** was synthesized via a transition metal-free strategy utilizing the Huisgen reaction (Scheme 24) (Arandhara et al., 2023). The process effectively employed *N*-alkynyl hydroxyisoindolinones **81** as primary substrates, which reacted with sodium azide in DMF at 130 °C, facilitated by BF_3_·OEt_2_.

The reaction mechanism initiates with the *in situ* generation of *N*-acyliminium ion intermediates **I** from amido alcohol substrates **81** under Lewis acidic conditions. These intermediates subsequently undergo nucleophilic attack by azide ions, forming azide-substituted intermediates **III** that proceed through intramolecular [3 + 2] cycloaddition (Huisgen reaction) to construct the seven-membered diazepine ring. The resultant seven-membered ring compounds were obtained in moderate yields and demonstrated excellent compatibility with a wide array of substituents, including aromatic, aliphatic, and heterocyclic groups on the isoindolinone scaffold, underscoring the adaptability of this synthetic approach.

#### 2.2.2 C–C fused

In addition to the N–C fused pyrrolo [1,3]diazepine derivatives, the C–C fused analogs represent another important class of regioisomeric frameworks, in which the pyrrole and 1,3-diazepine rings are linked via direct carbon–carbon bonds. Among these structurally distinct scaffolds, pyrrolo[2,3-e][1,3]diazepine has emerged as a unique and valuable subtype, particularly for the development of antibacterial agents targeting drug-resistant pathogens. The representative synthetic route toward this privileged scaffold is summarized below.

##### 2.2.2.1 Pyrrolo[2,3-e][1,3]diazepine

**SCHEME 25:**
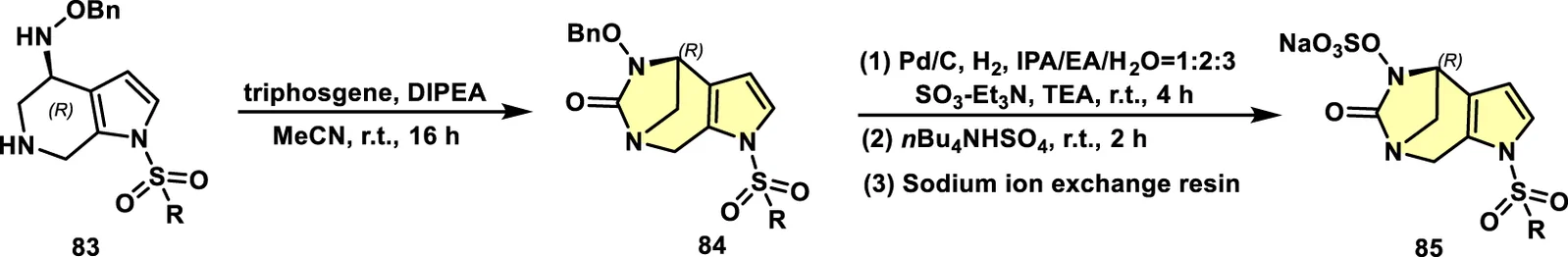
Synthesis of urea-containing tricyclic compound 85 as β-lactamase inhibitor.

As a distinct subclass of C–C fused 1,3-diazepine derivatives, urea-containing pyrrolo[2,3-e][1,3]diazepines have emerged as innovative scaffolds for combating antibacterial resistance. A patent filed by Chengdu Tetrahedral Drug Research Co., Ltd. has disclosed a novel class of urea-containing tricyclic β-lactamase inhibitors with a pyrrolodiazepine-derived scaffold, marking a significant structural advancement in overcoming bacterial resistance (Scheme 25) (Yu et al., 2025). The core compounds are characterized by a unique tricyclic framework integrated with a urea moiety, which enhances binding affinity. The synthesis was accomplished through a series of critical steps, including cyclization, debenzylation, sulfonation, and ion exchange.

Initially, compound **83** underwent a triphosgene-mediated ring-closing reaction in acetonitrile, using DIPEA as the base, to construct the tricyclic urea-containing scaffold **84**. Subsequent palladium-catalyzed hydrogenation facilitated debenzylation, while sulfonation with a triethylamine sulfur trioxide complex introduced the essential sulfonate moiety. Finally, treatment with a sodium ion exchange resin yielded the target compound **85** as a white solid.

### 2.3 Methods for the synthesis of 1,2-diazepines fused pyrrole

Following the discussion of 1,4- and 1,3-diazepine-fused pyrrole derivatives, 1,2-diazepine-fused pyrrole scaffolds represent the least extensively studied subclass, but their distinctive ring connectivity and versatile reactivity render them valuable building blocks for the construction of novel bioactive heterocyclic architectures. Among these promising frameworks, N–C fused pyrrolo[1,2-b][1,2]diazepines stand out as a key subtype, and their efficient synthetic approaches are summarized below.

#### 2.3.1 N–C fused

##### 2.3.1.1 Pyrrolo[1,2-b][1,2]diazepine

**SCHEME 26:**
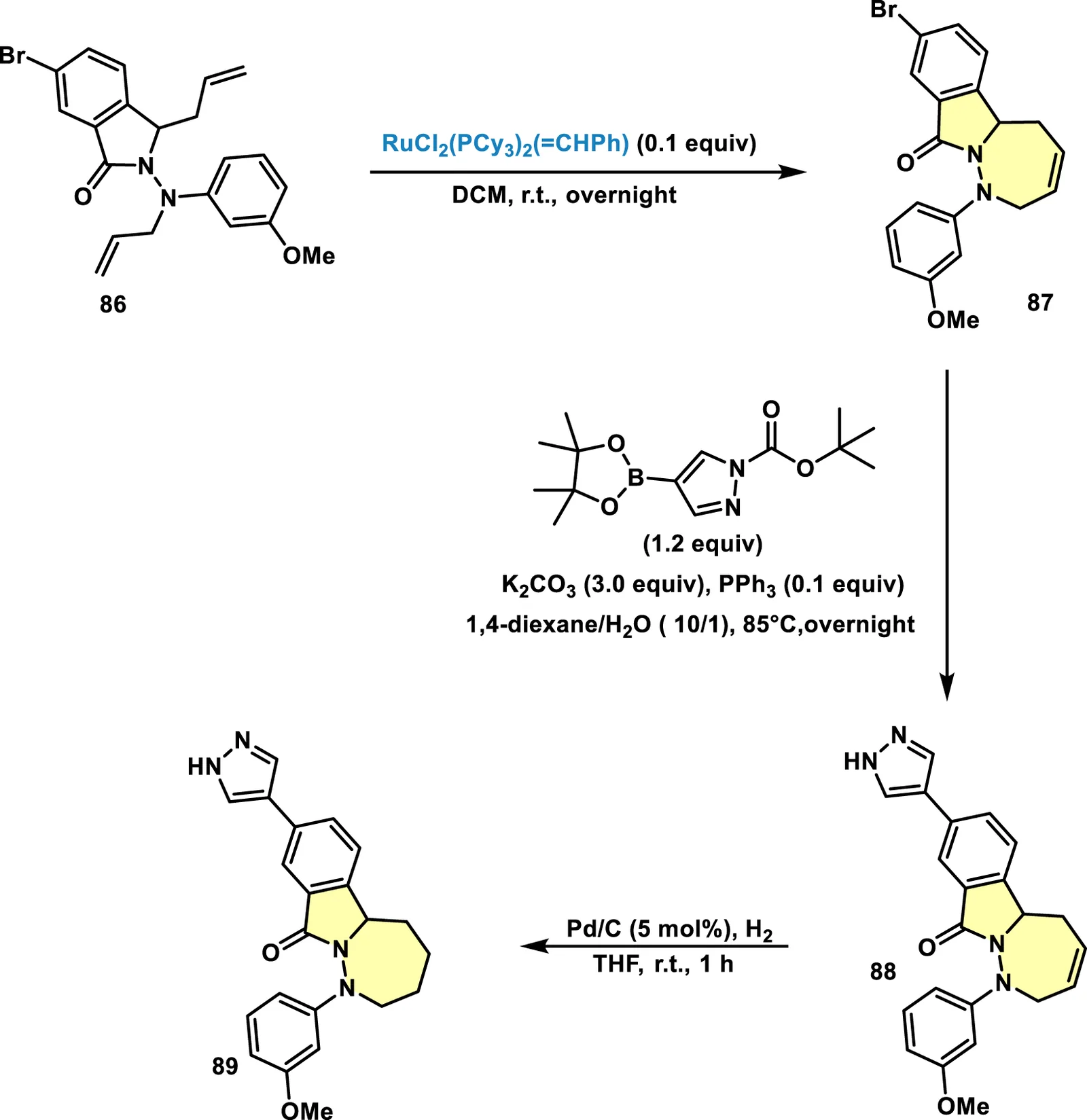
Synthesis of GRK2 inhibitors.

Finally, 1,2-diazepine-fused pyrrole derivatives constitute a relatively underexplored yet equally significant subclass, characterized by unique ring connectivity and remarkable affinity for biological targets. The synthesis of two novel fused isoindolin-1-one derivatives, designated as compounds **88** and **89**, incorporating a diazepino-fused scaffold, was accomplished as delineated in the international patent WO2020212434 (Scheme 26) (Xu, 2020). The synthetic strategy was meticulously devised, with ring-closing metathesis serving as the central step. The synthesis initiated with the treatment of a diallylated intermediate **86** using the first Generation Grubbs Catalyst, which successfully established the core cyclic structure. Subsequently, a Suzuki-Miyaura coupling reaction was employed to introduce the pyrazol-4-yl moiety, resulting in the formation of the unsaturated derivative **88**. To synthesize the saturated analogue **89**, compound **88** underwent a hydrogenation step. This transformation was catalyzed by Pd/C, effectively saturating the cyclic alkene present in the structure of compound 16, thereby yielding the final product **89**.

This paper reviews the advancements in the synthesis of pyrrolodiazepine derivatives over the past 5 years. The synthetic strategies are categorized into seven primary groups based on reaction types, catalytic systems, and cyclization mechanisms, as detailed in Table 1. Each strategy presents distinct synthetic benefits and applicable contexts, alongside certain limitations.

**TABLE 1:** Comparison of synthetic strategies for pyrrolodiazepine derivatives.

Synthetic method	Core advantages	Main limitations	Regioselectivity profile	Scalability (large-scale production)	Sustainability (green chemistry)	Representative skeleton types
Base-mediated cyclization reaction	Simple mechanism, convenient operation, no noble metal requirement, readily available starting materials	Strong alkaline/high temperature prone to side reactions, poor regioselectivity for some substrates, low efficiency for sterically hindered substrates	Mature control for 1,7- and 1,2-fused modes, highly dependent on substrate substituents	Easy to scale up (readily available reagents, simple operation)	Moderate (inorganic salt waste, no noble metal residue)	Pyrrolo[3,2-e][1,4]diazepine, Pyrrolo[2,3-e][1,4]diazepine
Transition-metal-catalyzed cyclization reaction (Pd/Au/Cu/Rh)	High catalytic activity, mild reaction conditions, precise regioselectivity/stereoselectivity, excellent functional group tolerance	High cost of noble metal catalysts, some require complex ligands/additives, sensitive to water and oxygen	Precise control of regioselectivity for all fused modes, high enantioselectivity achievable with chiral ligands	Scalable, requires optimization of catalyst cost and ligand recovery	Moderate (noble metal residue, ligand waste)	Pyrrolo[1,2-a][1,4]diazepine, Pyrrolo[1,2-day][1,4]diazepine, geminal dipyrrolodiazepine
Cascade/domino cyclization reaction	High step economy, no intermediate isolation required, convenient operation, excellent atom economy	Difficult reaction design, challenging selectivity control for multi-step reactions, narrow substrate scope for some reactions	High regioselectivity achievable via rational reaction design, highly dependent on substrate structure	Easy to scale up, requires optimization of reaction condition stability	Good (low solvent consumption, few side products)	Pyrrolo[2,3-e][1,4]diazepine, geminal dipyrrolo[1,2-a:2′,1′-c][1,4]benzodiazepine
Multicomponent reaction (MCR)	High modularity, rich molecular diversity, convenient operation, excellent atom economy	Challenging selectivity control for some reactions, prone to side products, high requirement for condition optimization	High regioselectivity achievable via substrate design, sensitive to reaction conditions	Easy to scale up, suitable for rapid compound library construction	Good (one-pot process, low solvent consumption)	Pyrrolo[3,4-e][1,4]diazepine, pyrrole-fused triazolobenzodiazepine
Photoredox catalytic reaction	Mild reaction conditions, no high temperature/strong acid/base required, environmental friendliness, excellent functional group tolerance	Some require noble metal photosensitizers, sensitive to oxygen, high operation requirements	Enables regioselectivity difficult to achieve via traditional methods through radical pathways	Scalable, requires optimization of light source and photosensitizer cost	Excellent (room temperature, no strong acid/base, few side products)	Pyrrolo[1,2-d][1,4]diazepinone, highly functionalized pyrrolodiazepinone
Organocatalytic cyclization reaction	Readily available catalysts, no metal residue, mild reaction conditions, controllable stereoselectivity	High catalyst loading, some require high temperature, long reaction time, low efficiency for sterically hindered substrates	High stereoselectivity achievable via organic catalysts, regioselectivity highly dependent on substrate	Easy to scale up, low catalyst cost	Excellent (no metal, no toxic reagents)	Pyrrolo[1,2-a][1,4]diazepine, Pyrrolo[2,1-c][1,4]diazepine
Green synthetic strategy (bio-based reagents/ultrasound/ionic liquid)	Environmental friendliness, recyclable reagents, convenient operation, compliant with green chemistry principles	Narrow substrate scope for some reactions, high cost of bio-based reagents	High regioselectivity achievable via synergistic catalysis, highly dependent on substrate structure	Scalable, ionic liquid recyclability reduces cost	Excellent (bio-based reagents, recyclable catalysts, low toxic waste)	Pyrrolo[1,2-a][1,4]diazepinone, pyrrolobenzodiazepine-triazole hybrid

Base-mediated cyclization is identified as the most traditional and fundamental strategy for constructing pyrrolodiazepine frameworks. This approach focuses on the efficient formation of the seven-membered diazepine ring through intra- or intermolecular nucleophilic addition, elimination, and cyclization processes facilitated by inorganic or organic bases. The principal advantages of this method include a straightforward reaction mechanism, ease of operation, the absence of a requirement for noble metal catalysts, and the availability of starting materials, making it the preferred choice for laboratory-scale synthesis of target compounds (Figueras, 2004; Islam et al., 2025; Shi et al., 2023). For instance, Belal et al. successfully synthesized pyrrolo[3,2-e][1,4]diazepine derivatives via a potassium carbonate-promoted intramolecular cyclization, followed by *N*-alkylation to finalize the preparation of the target derivatives (Belal et al., 2022). This methodology is characterized by mild reaction conditions and a high tolerance for various functional groups.

Nonetheless, this reaction type exhibits notable limitations: strongly alkaline environments and elevated temperatures can lead to side reactions, controlling regioselectivity in certain substrates poses challenges, and both reaction efficiency and selectivity significantly diminish for substrates with substantial steric hindrance. Furthermore, the majority of base-mediated cyclization reactions necessitate multiple operational steps, which results in relatively low atom and step economy, thereby restricting their applicability in constructing complex polycyclic frameworks.

Transition-metal catalysis represents the predominant and extensively utilized synthetic approach for the construction of pyrrolodiazepine frameworks. The fundamental objective is to facilitate the efficient and highly selective formation of C–C and C–N bonds through transition metal catalysts such as Pd, Au, Cu, and Rh. This approach addresses the challenges of regioselectivity and efficiency inherent in traditional base-mediated reactions. The principal advantages of transition-metal-catalyzed reactions include high catalytic activity, mild reaction conditions, precise control over regioselectivity and stereoselectivity, and exceptional tolerance to functional groups (Kim et al., 2020; Sakthivel et al., 2023; Sinha et al., 2023). These attributes enable the efficient synthesis of complex polycyclic structures that are challenging to achieve using conventional methods.

Among the various catalytic systems, the palladium-based catalytic system exhibits the broadest range of applications, encompassing diverse reaction types such as carbonylative cyclization, aminoalkylative cyclization, and C–H bond activation cyclization. For instance, Shi et al. successfully synthesized pyrrolo[1,2-a][1,4]diazepinone derivatives via a palladium-catalyzed tandem carbonylative aza-Wacker-type cyclization reaction, achieving yields of the target product up to 90% and demonstrating good compatibility with substrates possessing different substituents (Shi et al., 2021). Furthermore, the palladium-catalyzed three-component aminoalkylative cyclization reaction enables the highly selective synthesis of pyrrolodiazepine derivatives containing exocyclic diene structures, with the products exhibiting complete *E*-selectivity (Zou et al., 2023b). This provides abundant reaction sites for subsequent structural derivatization.

The gold-based catalytic system offers distinct advantages in the activation and cyclization of alkynes and allenes, enabling highly selective seven-endo cyclization through precise modulation of electronic and steric effects. For example, Au(I)-catalyzed hydroarylation of allenes efficiently assembles pyrrolo[1,2-d][1,4]diazepine frameworks. Notably, this method achieves up to 96% ee, furnishing an effective approach to chiral pyrrolodiazepine derivatives (Zhou et al., 2025).

However, transition-metal-catalyzed reactions suffer from several practical limitations. Noble metal catalysts are costly, complex ligands, oxidants, or additives are often required, and the reaction systems are sensitive to moisture and oxygen, necessitating stringent operational conditions. Additionally, the substrate scope of certain metal-catalyzed reactions is restricted, with reaction efficiency diminishing for substrates containing strong electron-withdrawing groups.

The cascade or domino cyclization reaction represents the most step-economical synthetic strategy for the construction of pyrrolodiazepine frameworks. This strategy integrates multiple sequential reactions in a single vessel, eliminating intermediate isolation. Complex structures are thus assembled in one step, significantly improving synthetic efficiency. The primary advantages of this reaction type include high step economy, operational simplicity, the avoidance of isolating unstable intermediates, minimal side reactions, and superior atom economy (Liao et al., 2021; Shivam et al., 2022). These attributes make it a focal point of current research in the synthesis of complex heterocyclic compounds.

For instance, Poletto et al. successfully demonstrated the efficient one-step construction of pyrrolo[2,3-e][1,4]diazepine frameworks via a cascade cyclization/annulation reaction, employing β-enaminodione and o-phenylenediamine as starting materials under metal-free conditions (Poletto et al., 2024). This process involves a continuous sequence of intramolecular cyclization, iminium ion formation, and intermolecular annulation. The target product can be isolated and purified through simple filtration and washing, underscoring the method’s operational convenience. Furthermore, a domino reaction enables the one-step synthesis of intricate polycyclic frameworks, such as geminal dipyrrolodiazepine derivatives, at ambient temperature, achieving a yield of 78%. This exemplifies the efficacy of cascade reactions in constructing complex molecular architectures.

However, the limitations of such reactions are primarily evident in several aspects: the complexity of reaction design, the necessity for precise control over the rate and selectivity of multiple reactions, the restricted substrate scope for certain reactions, and diminished reaction efficiency and selectivity when dealing with substrates possessing significant steric hindrance. Additionally, some cascade reactions necessitate the use of strong acids or bases as catalysts, which can limit the tolerance for various functional groups.

The multicomponent reaction represents a highly modular and versatile synthetic approach for the construction of pyrrolodiazepine frameworks. This method involves the simultaneous chemical transformation of three or more starting materials within a single reaction system, enabling the one-step synthesis of complex heterocyclic structures. Such an approach facilitates the rapid generation of compound libraries and enhances molecular diversity, which is crucial for drug screening processes. The principal advantages of multicomponent reactions include their high modularity, extensive molecular diversity, operational simplicity, superior atom economy, and the capacity to swiftly generate structurally diverse compound libraries (Buskes et al., 2023; John et al., 2021; Mohlala et al., 2024). These attributes position multicomponent reactions as a pivotal synthetic strategy for lead compound discovery in medicinal chemistry.

One representative example relies on a three-component one-pot protocol. With *p*-toluenesulfonic acid as the catalyst, tetramic acids, *o*-phenylenediamine and aromatic aldehydes directly react to furnish pyrrolidinone-substituted pyrrolo[3,4-e][1,4]diazepines in one step with high efficiency (Han et al., 2021). The yield of the desired product ranged from 80% to 88%, and the diastereoselectivity achieved was greater than 20:1 d.r. This reaction exhibits excellent compatibility with aromatic aldehydes possessing various substituents, facilitating the rapid construction of a library of structurally diverse derivatives. Furthermore, through isocyanide-based multicomponent reactions, the efficient synthesis of pyrrole-fused triazolobenzodiazepine derivatives can be realized under solvent-free and catalyst-free conditions, with product yields ranging from 72% to 91% (Norouzi et al., 2024). This outcome underscores the significant advantages of multicomponent reactions.

The limitations of this type of reaction are mainly reflected in: the selectivity control of some multicomponent reactions is difficult, side reactions are prone to occur, and the optimization of reaction conditions is highly required. In addition, the substrate scope of some multicomponent reactions is limited, and the reaction efficiency decreases for substrates bearing strong electron-withdrawing groups or large steric hindrance substituents.

Photoredox catalysis has emerged as a promising green synthetic strategy in recent years (Cabanero and Rovis, 2025; Coppola et al., 2022; Ghosh et al., 2023). Central to this approach is the generation of radical intermediates via a single electron transfer process under visible light irradiation, facilitating cyclization reactions that are challenging to achieve using conventional methods. This innovation offers a novel approach for constructing pyrrolodiazepine skeletons. Key advantages of this approach include mild reaction conditions, broad functional group tolerance, and environmental sustainability. Notably, it avoids high temperatures, strong acids or bases, and noble metal catalysts. These attributes align with the principles of green chemistry.

Brambilla et al. successfully synthesized pyrrolo[1,2-d][1,4]diazepinone derivatives via a photoredox-catalyzed cascade cyclization reaction. This process employed *N*-(2-(1H-pyrrol-1-yl)phenyl)-*N*-benzylmethacrylamide and aroyl chlorides as starting materials, under visible light irradiation, facilitating a continuous sequence of acyl radical addition and intramolecular cyclization. Notably, the reaction proceeded efficiently at ambient temperature, achieving a product yield of 57% with a diastereoselectivity ratio of 4:1 (Brambilla et al., 2023). The limitations of this type of reaction are mainly reflected in: some photocatalytic reactions require the use of expensive noble metal photosensitizers such as iridium and ruthenium, the reaction system is sensitive to oxygen, and the operation requirements are relatively high. In addition, the substrate scope of some photocatalytic reactions is limited, and the reaction efficiency decreases for substrates bearing strong electron-withdrawing groups.

Organocatalysis represents a significant complementary approach for the synthesis of pyrrolodiazepine frameworks. The primary objective is to facilitate efficient and highly selective reactions using small organic molecule catalysts, such as amines, phosphines, and carbenes, thereby circumventing the need for metal catalysts. This aligns with the principles of green chemistry. The principal benefits of this methodology include the availability of catalysts, mild reaction conditions, absence of metal residues, environmental sustainability, and controllable stereoselectivity, rendering it highly valuable in pharmaceutical synthesis. For instance, the DABCO-catalyzed [4+3] cyclization reaction employing *o*-methoxyphenyl-substituted pyrrole-2-carboxamide and MBH carbonate as starting materials successfully yielded pyrrolo[1,2-a][1,4]diazepine derivatives. The resultant product exhibited complete *trans* stereoselectivity (d.r. > 20:1) with yields reaching up to 67% (Wang et al., 2025). Furthermore, the DBU-catalyzed one-pot three-component reaction facilitated the efficient synthesis of pyrrolodiazepine derivatives at 100 °C, achieving yields of up to 91%, thereby underscoring the advantages of this approach (Lee et al., 2025).

The limitations associated with this type of reaction are primarily evident in several aspects: the typically high dosage of organic catalysts required, the necessity for elevated temperature conditions in certain reactions, prolonged reaction times, and diminished reaction efficiency and selectivity when dealing with substrates that exhibit significant steric hindrance. Furthermore, the substrate scope of organocatalytic reactions is relatively constrained, with reduced reactivity observed for substrates containing strong electron-withdrawing groups.

Green synthesis represents a significant trajectory in the contemporary field of pyrrolodiazepine synthesis. The central goal is to minimize organic solvents and toxic reagents. This is achieved through green synthetic methodologies such as bio-based reducing agents, recyclable catalysts, ultrasound assistance, and ionic liquids. These strategies are environmentally benign, allow reagent recycling and feature simple operation. All these advantages comply with green chemistry principles, making such approaches a core research direction for pharmaceutical synthesis in the future.

For instance, the glucose-mediated nitro-reductive cyclization reaction, utilizing D-glucose as a bio-based reducing agent, facilitated the efficient synthesis of pyrrolo[1,2-a][1,4]diazepinone derivatives in a mixed solvent system of DMSO:H_2_O (1:1) (Panday et al., 2024). This approach yielded the target product with an 80% efficiency, thereby circumventing the need for toxic metal reducing agents typically employed in conventional nitro reduction reactions. Moreover, an ultrasound-assisted, ionic liquid-molecular iodine synergistically catalyzed domino reaction enabled the efficient synthesis of pyrrolobenzodiazepine-triazole hybrids. The ionic liquid demonstrated recyclability for up to four cycles without significant loss in yield, and the reaction proceeded at 40 °C, achieving a product yield of up to 90% (Saquib et al., 2023). This underscores the advantages of green synthetic methodologies. However, the limitations of such strategies are primarily associated with a restricted substrate scope, as reaction efficiency diminishes for substrates with strong electron-withdrawing groups or significant steric hindrance. Additionally, the relatively high cost of certain bio-based reagents poses a barrier to their large-scale application.

### 2.4 Conformational flexibility and dynamics of pyrrolodiazepine scaffolds

After summarizing diverse synthetic methodologies for constructing various pyrrolodiazepine regioisomers with distinct ring fusion modes, it is essential to discuss their intrinsic conformational properties. The seven-membered diazepine ring exhibits unique structural flexibility, and the fusion of pyrrole ring further modulates the overall molecular conformation, which profoundly determines the interactions between pyrrolodiazepine derivatives and biological targets.

Seven-membered diazepine rings are well known for their intrinsic conformational flexibility, which distinguishes them from rigid five-membered pyrrole and planar six-membered aromatic heterocycles (Valenti et al., 2021). Owing to moderate ring strain and free rotation of C–C and C–N single bonds, diazepine skeletons can adopt multiple low-energy conformers that dynamically interconvert in solution and physiological environments (Leśniewska and Przybylski, 2024). Among the three main diazepine isomers, 1,4-diazepine exhibits the highest conformational freedom, making it the most widely used parent ring in current pyrrolodiazepine research, while 1,3- and 1,2-diazepines show relatively constrained conformations due to the adjacent distribution of nitrogen atoms (Jarończyk et al., 2023; Zhou et al., 2026).

The ring fusion pattern between pyrrole and diazepine serves as the dominant factor regulating the overall molecular conformation. For C–C fused pyrrolodiazepines such as pyrrolo[3,2-e][1,4]diazepines, the continuous aromatic π-conjugation restricts the flexibility of the seven-membered ring and forms a quasi-planar molecular geometry. This planar structure is highly compatible with the planar active sites of kinases and nucleic acids, which accounts for the excellent antiproliferative and kinase inhibitory activities of these derivatives (Belal et al., 2022). In contrast, N–C fused pyrrolodiazepines represented by pyrrolo[1,2-a][1,4]diazepines possess a bridgehead nitrogen atom, which breaks the conjugated system and forces the molecule into a folded three-dimensional conformation. The retained flexibility of the diazepine ring enables dynamic conformational switching, allowing these compounds to fit the stereoscopic binding cavities of GPCRs, ion channels and hydrolases, thus exerting potent central nervous system regulatory and antimicrobial effects (Obaji et al., 2024; Picconi et al., 2022).

Substituents and stereocenters further modulate conformational behaviors. Small alkyl and alkoxy groups barely alter the preferred conformation, while bulky aryl and halogenated aromatic substituents introduce steric hindrance to lock specific conformers, improving target selectivity (Basilaia et al., 2022). Chiral centers generated during asymmetric synthesis also fix molecules into exclusive spatial configurations, resulting in distinct pharmacological performance between enantiomers and diastereomers (McVicker and O’Boyle, 2024). Notably, the photoswitchable pyrrolodiazepine derivative with an azobenzene group achieves reversible conformational rearrangement under different wavelengths of light. The *cis* and *trans* conformers show dramatically different residence time on vasopressin V_2_ receptor, demonstrating the great potential of conformational regulation in precision pharmacology (Gu et al., 2023).

Conformational properties directly govern molecular recognition and druggability. Moderate flexibility facilitates induced-fit binding to biological targets, while enhanced rigidity reduces non-specific interactions and improves selectivity (Basu and Fletcher, 2026). At present, most studies on pyrrolodiazepines focus on synthesis and biological evaluation, and systematic investigations on conformational dynamics via X-ray crystallography, NMR and molecular dynamics simulation are still insufficient. Further exploration in this field will provide a solid structural basis for the rational design of next-generation pyrrolodiazepine drugs.

The differences in conformational flexibility and dynamics among various pyrrolodiazepine scaffolds lay a solid structural foundation for their diverse biological activities and structure–activity relationships, which are discussed in detail in the following section.

## 3 Biological activities

In addition to the significant advancements made in the modular and regioselective synthesis of pyrrolodiazepine frameworks, comprehensive pharmacological evaluations have revealed that these fused heterocyclic systems exhibit diverse and potent biological activities across a broad spectrum of disease-related targets. By interacting with key proteins such as G protein-coupled receptors, enzymes, ion channels, kinases, and transcription factors, pyrrolodiazepine derivatives demonstrate promising therapeutic potential in various clinical domains, including central nervous system disorders, cancer, infectious diseases, and inflammation. This section systematically summarizes and discusses the biological activities and structure–activity relationships of representative pyrrolodiazepine derivatives, organized according to major therapeutic indications.

### 3.1 CNS disorders

**FIGURE 3:**
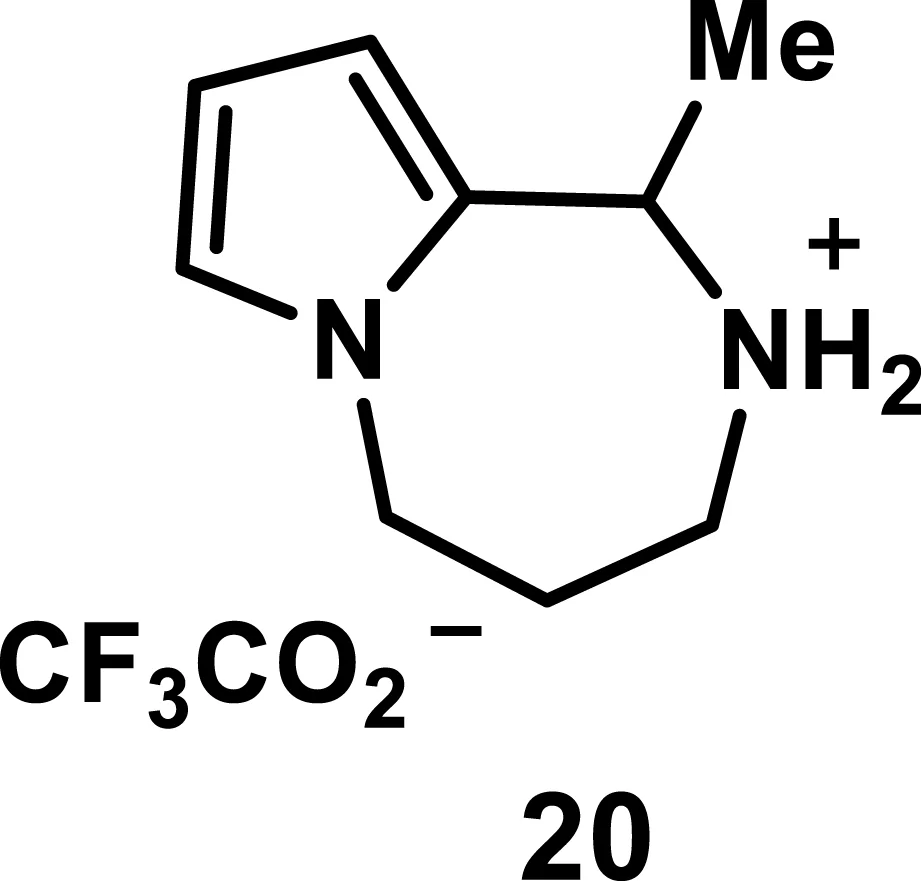
Structure of hTAAR1 agonist 20.

Pyrrolodiazepine derivatives have emerged as promising privileged scaffolds for the treatment of various CNS disorders, including psychiatric conditions, epilepsy, neuropathic pain, and neurodegenerative diseases. These compounds primarily exert their effects by targeting aminergic receptors, ion channels, and metabolic enzymes. The tetrahydropyrrolo [1,2-a]diazepine scaffold functions as a conformationally constrained mimic of tryptamine-derived trace amines, exhibiting potent agonistic activity towards the human Trace Amine-Associated Receptor 1 (hTAAR1) (Paramonova et al., 2024). Notably, compound **20** demonstrated an exceptional EC_50_ of 25 nM, comparable to the natural agonist tyramine, underscoring its potential for the treatment of schizophrenia and related mental disorders (Figure 3). SAR studies indicated that the presence of a small methyl substituent at the C2-position of the diazepine ring significantly enhanced receptor binding affinity, whereas the introduction of hydrophilic or bulky groups markedly diminished potency.

**FIGURE 4:**
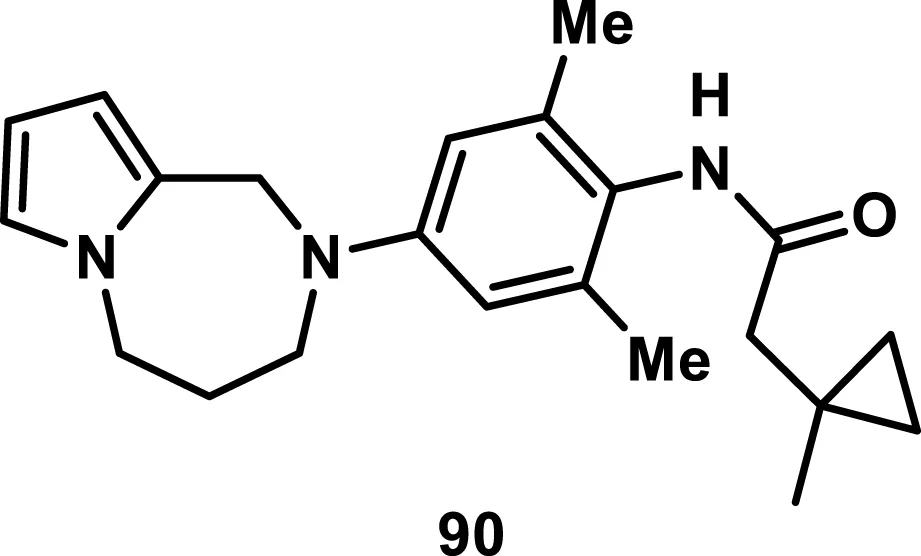
Structure of potassium channel regulator 90.

Furthermore, pyrrolodiazepine derivatives have been identified as potent agonists of the Kv7.2 (KCNQ2) channel, effectively suppressing neuronal hyperexcitability. Notably, compound **90** demonstrated significant agonistic activity with an EC_50_ of 3.7 μM, alongside enhanced subtype selectivity and permeability across the blood-brain barrier, thereby endorsing its potential for further development in the treatment of epilepsy, anxiety, and neuropathic pain (Figure 4) (Liang et al., 2023).

**FIGURE 5:**
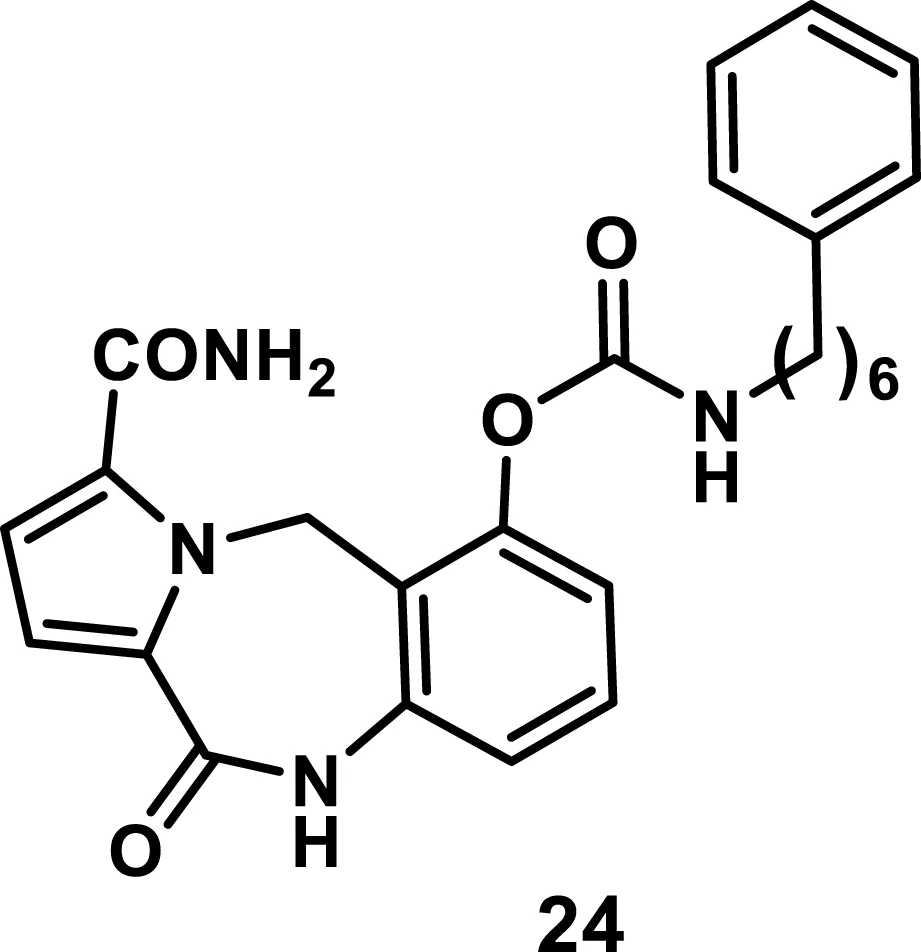
Structure of FAAH inhibitor 24.

Another critical central nervous system target of pyrrolodiazepine derivatives is fatty acid amide hydrolase (FAAH), a principal serine hydrolase involved in the degradation of endocannabinoids (Grillo et al., 2021). Compound **24**, a conformationally constrained pyrrolodiazepine analogue, exhibited potent and selective inhibition of FAAH with an IC_50_ of 35 nM in mouse brain tissue (Figure 5). It demonstrated no cross-reactivity with cannabinoid receptors CB1R/CB2R or monoacylglycerol lipase (MAGL), indicating remarkable target selectivity. In cellular models, compound **24** significantly inhibited LPS-induced NF-κB activation and exhibited no neurotoxicity at concentrations up to 50 μM, supporting its potential as an anti-inflammatory and neuroprotective agent for the treatment of temporal lobe epilepsy.

**FIGURE 6:**
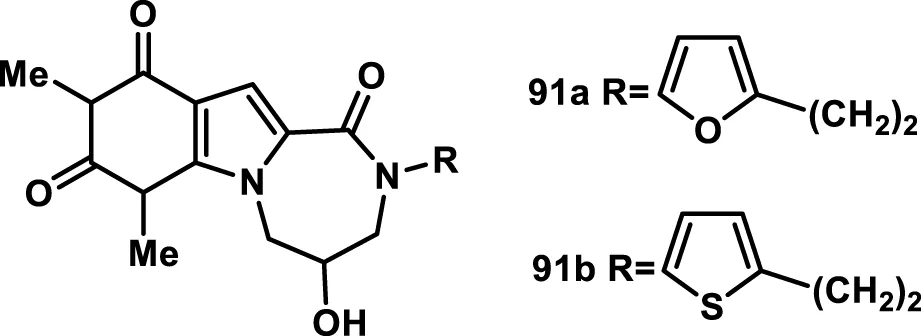
Structure of Adenosine A_2_A receptor antagonists 91.

Pyrrolodiazepine derivatives demonstrate significant potential in receptor pharmacology and computer-aided drug discovery. A series of pyrrole-fused 7-deazaxanthine derivatives **91** were engineered as adenosine A_2_A receptor antagonists utilizing machine learning techniques and molecular docking methodologies (Figure 6) (Muzychka et al., 2022). The selected compounds exhibited predicted Ki values ranging from one to 5 μM and demonstrated low acute toxicity in Daphnia magna, with 48-h LC_50_ values between 244.76 and 357.95 mg/L, thereby supporting their further development for the treatment of neurodegenerative diseases.

### 3.2 Anticancer activities

Pyrrolodiazepine derivatives exhibit significant antiproliferative effects across a wide range of human cancer cell lines through various mechanisms, including kinase inhibition, DNA binding, inhibition of BET bromodomains, and the reversal of multidrug resistance (MDR). Comprehensive structure–activity relationship studies have identified key structural features that determine anticancer efficacy and selectivity.

**FIGURE 7:**
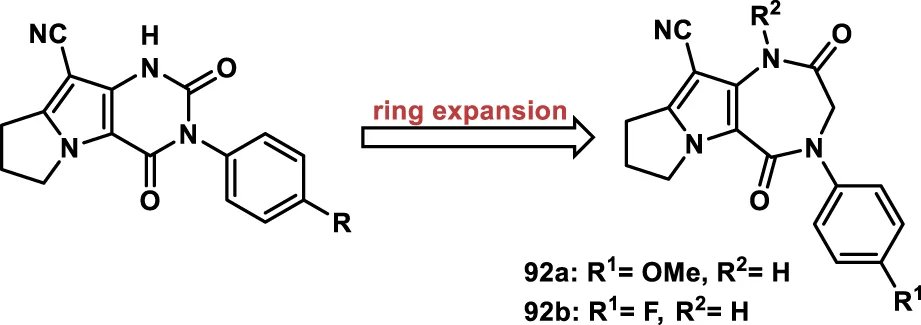
Design of P-glycoprotein inhibitors.

Pyrrolo[3,2-e][1,4]diazepine derivatives have been engineered as dual inhibitors of EGFR and CDK2, two well-established targets in cancer treatment (Figure 7). These compounds demonstrated potent antiproliferative activity against Hep3B, HCT116, and MCF-7 cancer cell lines, with IC_50_ values ranging from 0.154 to 0.840 μM. Further structural refinement through ring expansion from pyrimidine-fused analogs resulted in the development of compounds **92a** and **92b**, which function as P-glycoprotein (P-gp) inhibitors to counteract MDR in cancer chemotherapy. Notably, compound **92b** exhibited an IC_50_ of 0.19 μM against MCF-7 cells, with high selectivity indices compared to normal MRC5 fibroblasts, underscoring its potential for use in combination therapy (Belal et al., 2022; Shawky et al., 2021).

**FIGURE 8:**
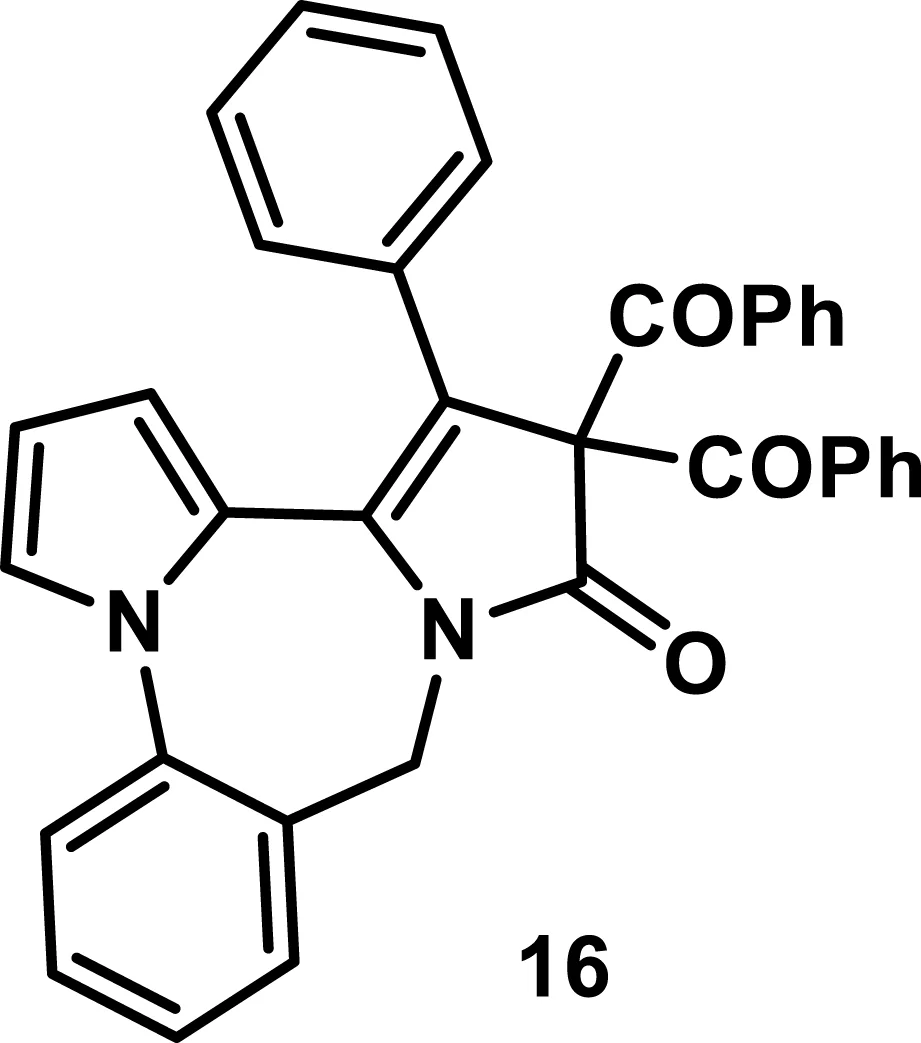
Structure of dipyrrolobenzodiazepine anticancer agent 16.

Geminal dipyrrolo[1,2-a:2′,1′-c][1,4]benzodiazepine (DPBD) derivatives were systematically evaluated for their cytotoxic effects on oral epidermal carcinoma (KB) and hepatocellular carcinoma (HepG2) cell lines. Among these, Compound **16** exhibited the highest cytotoxic potency, with IC_50_ values of 27.59 μg/mL for kB cells and 28.31 μg/mL for HepG2 cells (Figure 8). Structure-activity relationship (SAR) analyses indicated that benzoyl substitution significantly enhanced antiproliferative efficacy, whereas methoxy-substituted aromatic rings resulted in a loss of activity, underscoring the critical role of hydrophobic aromatic interactions in target binding (Zinoveva et al., 2025).

**FIGURE 9:**
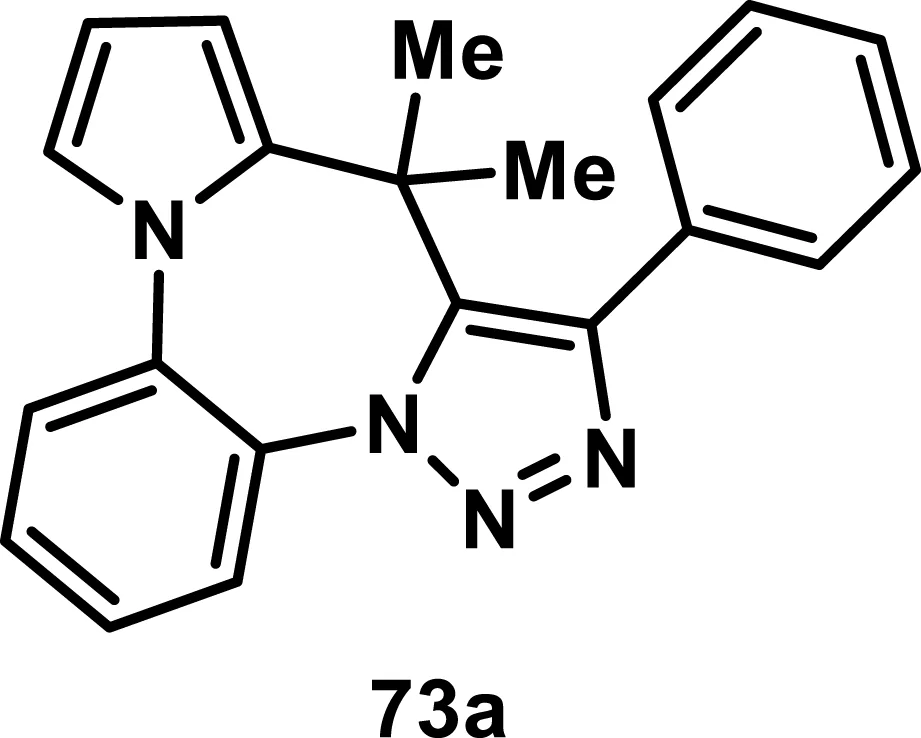
Structure of pyrrolobenzodiazepine–triazole anticancer agents.

Furthermore, pyrrolobenzodiazepine–triazole hybrid molecules were engineered using a molecular hybridization approach to consolidate anticancer pharmacophores within a single framework. These conjugates demonstrated potent and selective antiproliferative activity against breast, cervical, ovarian, lung, and colon cancer cell lines. The most effective derivative, compound **73a**, exhibited IC_50_ values ranging from 5.45 to 11.32 μM across seven cancer cell lines, while showing no significant toxicity towards normal HEK293 cells (IC_50_ > 20 μM) (Figure 9). SAR analysis further revealed that the presence of an unsubstituted phenyl group on the triazole moiety and gem-dimethyl substitution on the diazepine ring were crucial for achieving high potency (Saquib et al., 2023).

**FIGURE 10:**
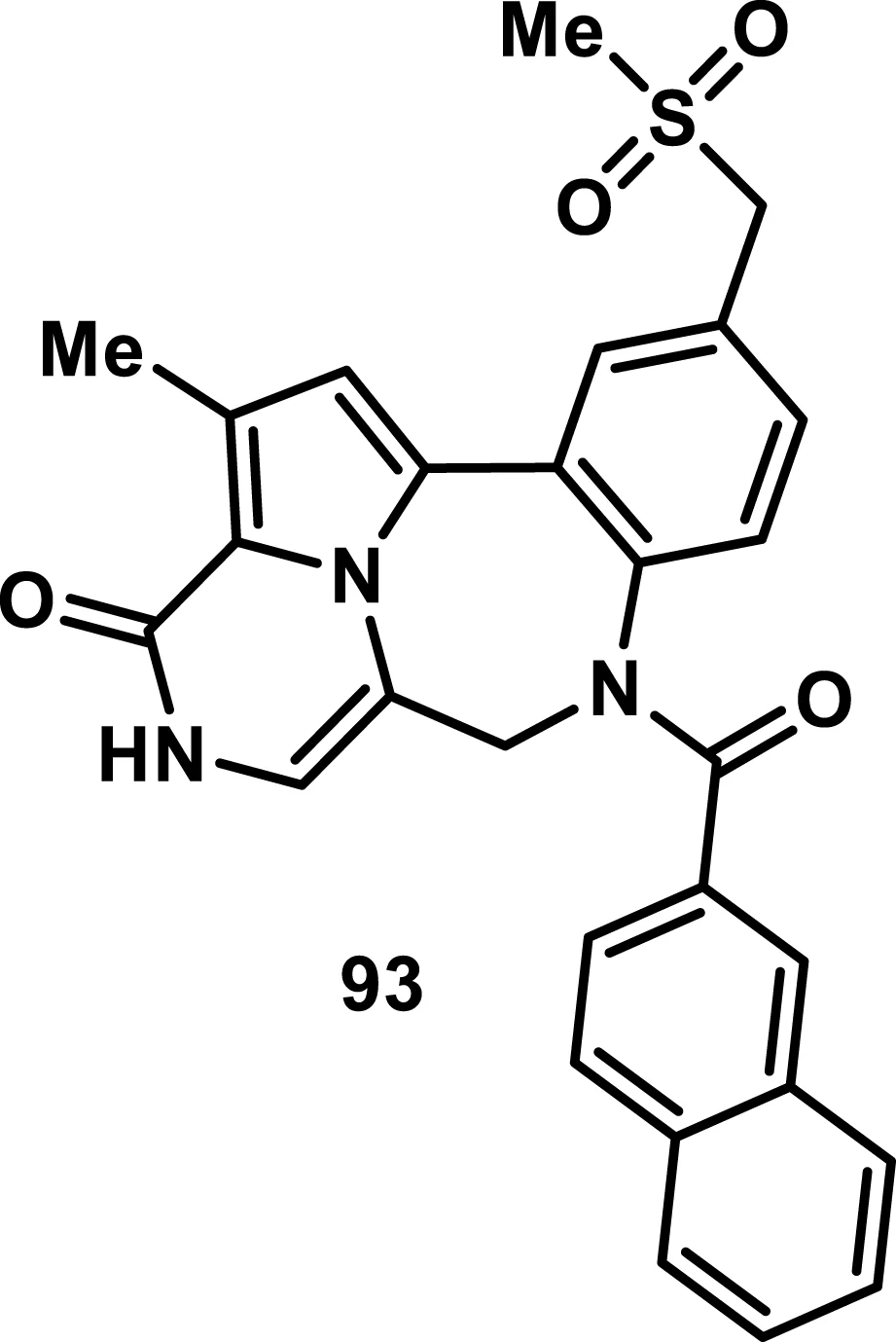
Structure of BET bromodomain inhibitor 93.

A highly potent tetracyclic pyrrolodiazepine, designated as compound **93**, has been developed as a selective inhibitor of BET bromodomains, exhibiting an IC_50_ of 1.6 nM for BRD4 BD2 (Figure 10). The rigidified scaffold of this compound optimally occupies the hydrophobic WPF shelf of BET bromodomains, resulting in exceptional binding affinity. In a murine model of LPS/D-GalN-induced acute liver injury, compound **93** significantly enhanced survival rates (84.6% at a dosage of 75 mg/kg), reduced serum ALT/AST levels, and suppressed the expression of pro-inflammatory cytokines, indicating its potential for the treatment of liver cancer and inflammation-related diseases (Chen et al., 2023).

### 3.3 Antimicrobial and antiviral activities

Pyrrolodiazepine derivatives demonstrate potent and selective activity against pathogenic bacteria, fungi, and viruses, particularly against drug-resistant strains, rendering them promising candidates for novel anti-infective agents.

**FIGURE 11:**
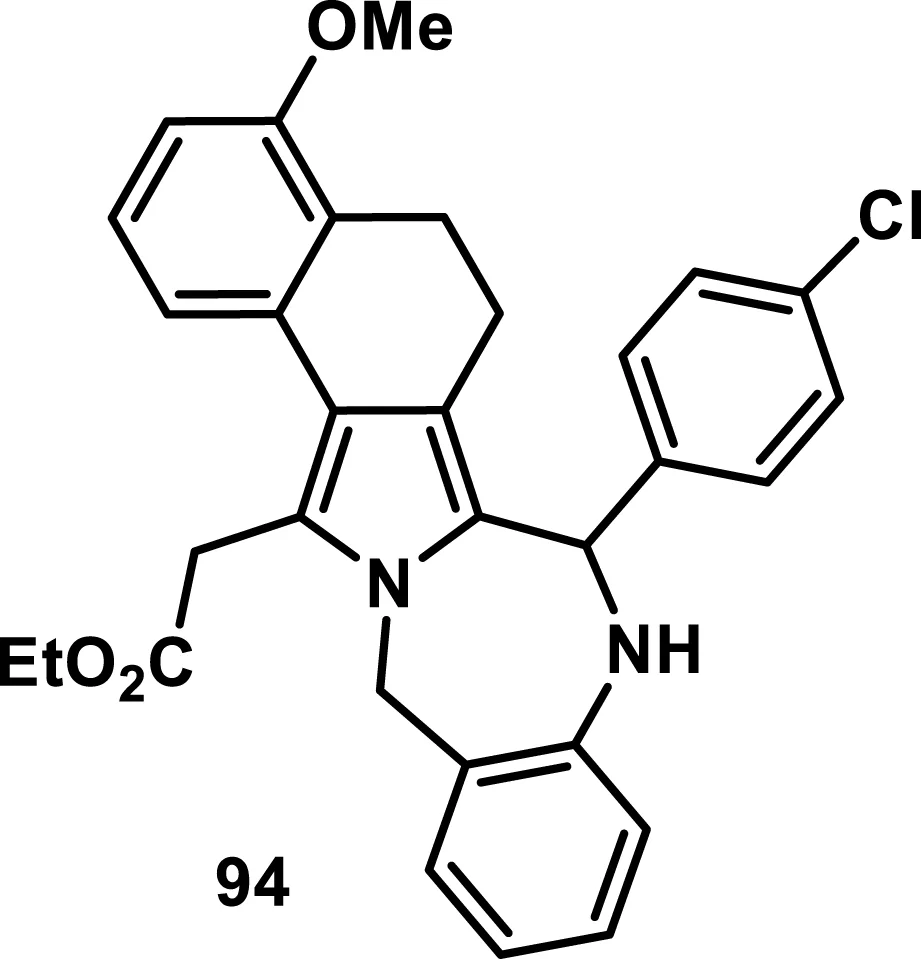
Structure of chlorophenyl pyrrolo benzodiazepine antibiotic.

A series of chlorophenyl-substituted pyrrolo[1,2-a][1,4]benzodiazepines demonstrated significant anti-MRSA activity. Notably, compound **94** exhibited minimum inhibitory concentration (MIC) values of 7.81 mg/L against both *Staphylococcus aureus* and *Staphylococcus* epidermidis, alongside a favorable therapeutic index (IC_50_ = 250 μg mL^-1^ in L929 fibroblasts) (Figure 11). Importantly, compound **94** effectively penetrated and eradicated preformed MRSA biofilms, representing a significant advantage over conventional antibiotics. SAR studies confirmed that the 4-chlorophenyl substituent was crucial for potent antibacterial activity (Mondal et al., 2024).

**FIGURE 12:**
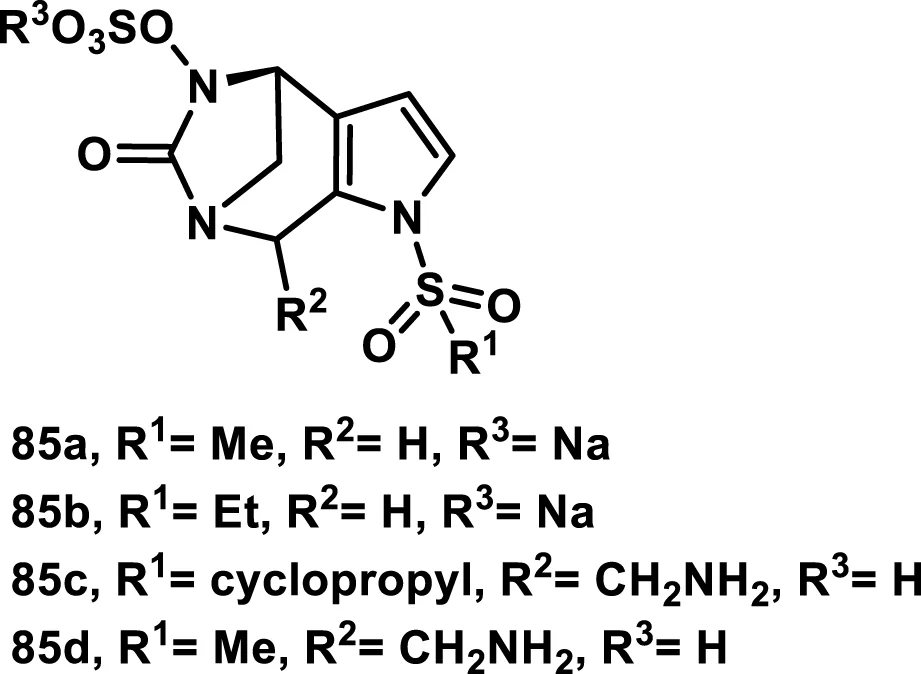
Structure of pyrrolo[2,3-e][1,3]diazepine antibacterial agent.

Additionally, pyrrolo[2,3-e][1,3]diazepine derivatives containing a urea moiety were identified as novel β-lactamase inhibitors capable of restoring the efficacy of ceftazidime, cefepime, and meropenem against multidrug-resistant Gram-negative strains producing TEM-1, KPC-2, AmpC, and NDM-1 enzymes (Figure 12). These compounds surpassed the clinically used avibactam, with MIC_50_ values ≤0.1 mg/L and IC_50_ ≤ 1 μM against class A/C/D β-lactamases, offering a promising new strategy to combat antimicrobial resistance (Yu et al., 2025).

**FIGURE 13:**
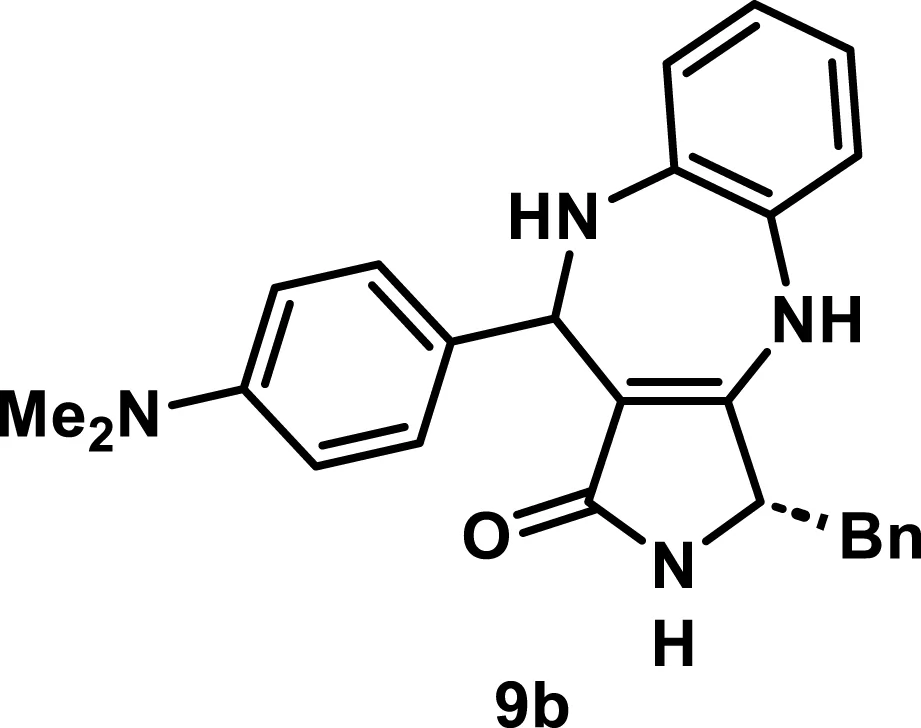
Structure of BVDV inhibitors.

In the field of antiviral research, pyrrolo[3,4-e][1,4]diazepine derivatives have demonstrated significant activity against the bovine viral diarrhea virus (BVDV), which serves as a surrogate model for the hepatitis C virus. Notably, compound **9b**, which features a dimethylamino group at the para position of the phenyl ring, exhibited an EC_50_ of 0.12 μM, making it approximately ten times more potent than ribavirin, while displaying no cytotoxicity in MDBK cells (Figure 13). SAR analysis revealed that electron-donating substituents at the para position of the phenyl ring substantially enhanced antiviral efficacy, whereas electron-withdrawing groups or halogens negated activity (Han et al., 2021).

### 3.4 Metabolic, renal and cardiovascular diseases

**FIGURE 14:**
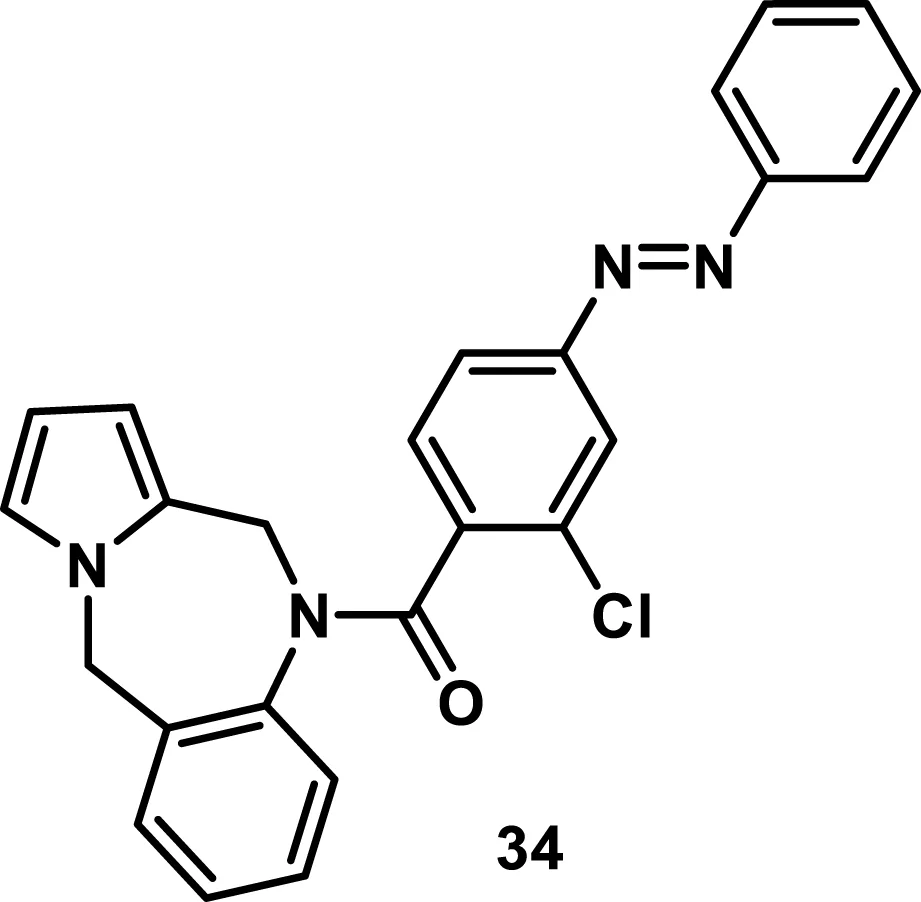
Structure of vasopressin V_2_ receptor antagonist.

Pyrrolodiazepine derivatives demonstrate promising pharmacological profiles for the treatment of metabolic, renal, and cardiovascular disorders by targeting GPCRs and serine/threonine kinases. Notably, a photopharmacological pyrrolodiazepine derivative **34** has been developed as a photoswitchable antagonist of the vasopressin V_2_ receptor (Figure 14). This compound undergoes dynamic interconversion between *trans* and *cis* isomers upon exposure to light at wavelengths of 365 nm and 435 nm, thereby enabling optical modulation of receptor residence time, ranging from 15 to 64 min. This spatiotemporal precision offers a novel therapeutic strategy for autosomal dominant polycystic kidney disease with reduced off-target effects (Gu et al., 2023).

**FIGURE 15:**
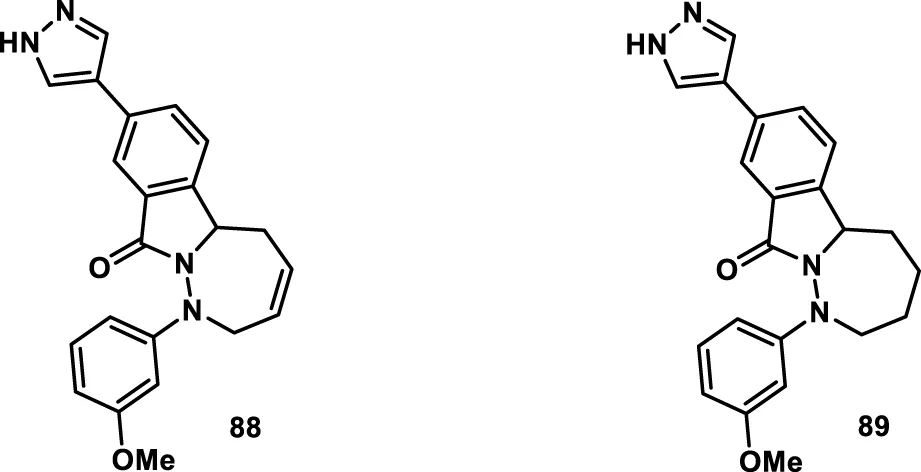
Structure of GRK2 inhibitors.

Additionally, pyrrolo[1,2-b][1,2]diazepine-fused isoindolinone derivatives **88** and **89** exhibit potent inhibitory activity against G protein-coupled receptor kinase 2 (GRK2), with IC_50_ values of 6 nM and 8 nM, respectively, representing an improvement of over 1000-fold compared to paroxetine (Figure 15). These compounds hold significant potential for the treatment of heart failure, type 2 diabetes, and non-alcoholic steatohepatitis (NASH) (Xu, 2020).

### 3.5 Structure–activity relationship

Despite the remarkable progress achieved in elucidating the biological activities of pyrrolodiazepines, the structure–activity relationship remains fragmented, with few studies systematically elucidating how fusion patterns, substituent types, and stereochemistry influence target interaction, potency, and selectivity. To address this critical gap, we herein provide an integrated analysis of the key structural determinants governing the pharmacological profiles of pyrrolodiazepine derivatives. Among these structural factors, substituent properties and substitution positions strongly affect molecular conformational stability, which is closely correlated with biological performance.

The conformational stability of pyrrolodiazepine scaffolds is profoundly governed by the nature and substitution position of pendant groups. The seven-membered diazepine ring inherently possesses high conformational flexibility and interconverts readily among planar, folded and twisted conformers. Substituents alter the energy barrier for conformational interconversion via steric hindrance, electronic effects and intramolecular noncovalent interactions, thereby stabilizing specific dominant conformations. In turn, these conformation preferences directly correlate with binding affinity, target selectivity and the overall biological performance of pyrrolodiazepine derivatives.

Steric hindrance is the most dominant factor modulating conformational profiles. Small alkyl substituents such as methyl groups installed at the saturated carbons of the diazepine ring slightly stabilize the folded conformations of N–C fused pyrrolo[1,2-a][1,4]diazepines while retaining intrinsic ring flexibility. This moderately constrained folded geometry well matches the binding cavities of GPCRs and ion channels, which accounts for the potent activity of compound **20** as a hTAAR1 agonist (Paramonova et al., 2024). In contrast, bulky aryl and halophenyl substituents introduce remarkable steric repulsion, locking C–C fused skeletons into rigid quasi-planar conformations. For instance, 4-chlorophenyl-substituted pyrrolo[1,2-a][1,4]benzodiazepine (compound **94**) adopts a highly stable planar conformation, which facilitates its penetration into bacterial membranes and contributes to excellent anti-MRSA activity (Mondal et al., 2024). Excessively bulky polycyclic moieties further rigidify the whole framework into twisted conformations, a structural feature critical for β-lactamase inhibition of compound **85** (Yu et al., 2025).

Electronic properties of substituents regulate π-conjugation and planar conformation stability across fused aromatic systems. Electron-donating groups including dimethylamino and methoxy strengthen delocalized π-electron systems and greatly enhance the stability of planar conformations. Compound **9b**, bearing a para-dimethylamino group on the aromatic ring, maintains a highly planar geometry and exhibits superior anti-BVDV activity, nearly ten times more potent than ribavirin (Han et al., 2021). Conversely, electron-withdrawing groups such as cyano weaken aromatic conjugation, disrupt molecular planarity and reduce the population of planar conformers, which compromises the antiproliferative potency of kinase inhibitors derived from pyrrolo[3,2-e][1,4]diazepine.

Polar functional groups capable of forming intramolecular hydrogen bonds serve as an effective tool to fix specific spatial orientations. The ortho-methoxyphenyl (OMP) directing group in pyrrolo[1,2-a][1,4]diazepine precursors forms a stable intramolecular hydrogen bond with the amide N–H bond (Wang et al., 2025). This interaction rigidifies the molecular skeleton exclusively into the trans-conformation (d.r. > 20:1) during [4+3] annulation, eliminating undesirable conformational isomers. Similarly, hydroxyl-substituted derivative **24** forms intramolecular hydrogen bonds with ring nitrogen atoms, stabilizing a compact folded conformation that leads to high selectivity and potent inhibitory activity against FAAH (Panday et al., 2024).

The substitution site further differentiates conformational behaviors. Substituents on the pyrrole ring mainly manipulate the planarity of conjugated C–C fused frameworks, while groups attached to the flexible seven-membered diazepine ring primarily tune folded or boat conformations of N–C fused isomers. Distal substitution on peripheral benzene rings exerts indirect effects through long-range steric and electronic modulation. Notably, the azobenzene moiety incorporated at the linker position of vasopressin V_2_ receptor antagonist (compound **34**) enables reversible photoisomerization between stable trans and cis conformations under different wavelengths of light (Gu et al., 2023). The two isomers display distinct conformational stabilities and receptor residence times (15 min for *trans* and 64 min for *cis*), achieving precise optical control over pharmacological activity.

Collectively, steric, electronic and noncovalent effects of substituents, combined with their substitution positions, jointly determine the dominant conformations of pyrrolodiazepines. A well-defined stable conformation is a prerequisite for optimal binding to biological targets, including kinases, enzymes, GPCRs and viral proteins. Clarifying such conformation–substituent–activity relationships provides rational guidance for the structural optimization of next-generation pyrrolodiazepine-based drug candidates.

Beyond substituent-induced conformational changes, ring fusion topology acts as the most fundamental structural parameter to shape molecular geometry and target selectivity. C–C fused pyrrolodiazepines, represented by pyrrolo[3,2-e][1,4]diazepines, favor planar geometries suitable for binding to kinase active sites and DNA-interacting targets, thereby exhibiting potent antiproliferative activity (Belal et al., 2022). In contrast, N–C fused analogs including pyrrolo[1,2-a][1,4]diazepines adopt folded three-dimensional architectures that match the binding pockets of GPCRs and enzymes, leading to preferred activity in CNS regulation, antimicrobial therapy, and receptor antagonism (Mondal et al., 2024; Paramonova et al., 2024; Zinoveva et al., 2024; Zinoveva et al., 2025). Meanwhile, the isomerism of the diazepine core (1,4-, 1,3-, 1,2-) further reshapes the spatial orientation of hydrogen-bond donors/acceptors and hydrophobic regions, resulting in distinct target recognition patterns.

Additionally, stereochemical configuration plays a critical role in target binding and bioactivity. The stereochemistry of pyrrolo[1,2-a][1,4]diazepines can significantly affect molecular conformation, binding orientation, and thus pharmacological outcomes. Rational stereochemical optimization helps improve binding affinity, subtype selectivity, and metabolic stability.

Pyrrolodiazepines exhibit a distinct three dimensional structure–activity relationship that dictates their druggable properties, which is governed by three synergistic core factors: ring fusion topology, substituent characteristics, and stereochemical configuration. The fusion mode defines the global pharmacophore geometry, substituent patterns fine-tune local binding interactions and physicochemical properties, and stereochemical rigidity minimizes the entropic cost of target association while maximizing selectivity. A holistic understanding of this interplay is indispensable for the rational design of next-generation pyrrolodiazepine therapeutics.

Nevertheless, the current SAR landscape remains fragmented; several underexplored regioisomeric classes (e.g., certain 1,3- and 1,2-diazepine fused systems) lack systematic profiling, and the impact of atropisomerism and dynamic conformational switching on *in vivo* efficacy has yet to be fully elucidated. Future investigations integrating high-resolution structural biology, computational alchemy, and advanced asymmetric synthesis are anticipated to decode these remaining complexities, ultimately accelerating the clinical translation of this privileged scaffold.

## 4 Conclusion and future prospects

Over the past five years, pyrrolodiazepines have established themselves as a pivotal scaffold in the field of medicinal chemistry, exhibiting significant versatility in synthetic methodologies and biological applications. This review systematically compiles recent advancements in the synthesis and therapeutic potential of these tricyclic heterocycles.

In terms of synthetic strategies, significant progress has been made in addressing the historical challenges of regioselectivity and step economy. We have classified the mainstream approaches into seven major categories (Table 1), highlighting the shift from traditional base-mediated cyclizations to more sophisticated catalytic systems. Transition-metal catalysis (particularly Pd and Au) has emerged as a dominant force, enabling precise control over stereochemistry and the construction of complex polycyclic frameworks such as geminal dipyrrolo[1,2-a:2′,1′-c][1,4]benzodiazepines. Concurrently, the emergence of green and sustainable synthetic methodologies reflects the field’s commitment to modern environmental standards. These approaches include organocatalysis, photoredox catalysis, and ultrasound-assisted reactions, and are typified by the application of biomass-derived reagents such as glucose.

Pharmacologically, pyrrolodiazepines have proven to be a treasure trove of bioactive agents. These compounds can modulate key targets in a wide range of therapeutic fields, including central nervous system diseases, oncology, and infectious diseases. Representative modulators include hTAAR1 agonists, Kv7.2 activators, BET bromodomain inhibitors, dual EGFR/CDK2 inhibitors, and novel β-lactamase inhibitors. This broad target profile underscores their excellent druggability. Notably, the development of photoswitchable derivatives, such as the vasopressin V_2_R antagonist, opens a new frontier in precision medicine, allowing for spatiotemporal control of drug activity.

Despite these strides, challenges remain. The SAR of many pyrrolodiazepine regioisomers is still not fully understood, and the synthetic access to certain underexplored isomers remains limited. Furthermore, while the biological potential is vast, the translation of these compounds into clinical candidates requires further optimization of pharmacokinetic properties and toxicity profiles.

Looking forward, the incorporation of artificial intelligence in drug design, alongside ongoing advancements in catalytic asymmetric synthesis, is projected to expedite the discovery of novel pyrrolodiazepine-based therapeutics. It is anticipated that this scaffold will persist as a prolific foundation for the development of next-generation pharmaceuticals, addressing unmet medical needs in oncology, neurology, and other fields.
